# Osteogenic differentiation of human mesenchymal stromal cells and fibroblasts differs depending on tissue origin and replicative senescence

**DOI:** 10.1038/s41598-021-91501-y

**Published:** 2021-06-07

**Authors:** Vera Grotheer, Nadine Skrynecki, Lisa Oezel, Joachim Windolf, Jan Grassmann

**Affiliations:** Clinic for Orthopedics and Trauma Surgery, Medical Faculty of the Heinrich Heine University, Moorenstr. 5, 40225 Düsseldorf, Germany

**Keywords:** Cell biology, Stem cells

## Abstract

The need for an autologous cell source for bone tissue engineering and medical applications has led researchers to explore multipotent mesenchymal stromal cells (MSC), which show stem cell plasticity, in various human tissues. However, MSC with different tissue origins vary in their biological properties and their capability for osteogenic differentiation. Furthermore, MSC-based therapies require large-scale ex vivo expansion, accompanied by cell type-specific replicative senescence, which affects osteogenic differentiation. To elucidate cell type-specific differences in the osteogenic differentiation potential and replicative senescence, we analysed the impact of BMP and TGF-β signaling in adipose-derived stromal cells (ASC), fibroblasts (FB), and dental pulp stromal cells (DSC). We used inhibitors of BMP and TGF-β signaling, such as SB431542, dorsomorphin and/or a supplemental addition of BMP-2. The expression of high-affinity binding receptors for BMP-2 and calcium deposition with alizarin red S were evaluated to assess osteogenic differentiation potential. Our study demonstrated that TGF-β signaling inhibits osteogenic differentiation of ASC, DSC and FB in the early cell culture passages. Moreover, DSC had the best osteogenic differentiation potential and an activation of BMP signaling with BMP-2 could further enhance this capacity. This phenomenon is likely due to an increased expression of activin receptor-like kinase-3 and -6. However, in DSC with replicative senescence (in cell culture passage 10), osteogenic differentiation sharply decreased, and the simultaneous use of BMP-2 and SB431542 did not result in further improvement of this process. In comparison, ASC retain a similar osteogenic differentiation potential regardless of whether they were in the early (cell culture passage 3) or later (cell culture passage 10) stages. Our study elucidated that ASC, DSC, and FB vary functionally in their osteogenic differentiation, depending on their tissue origin and replicative senescence. Therefore, our study provides important insights for cell-based therapies to optimize prospective bone tissue engineering strategies.

## Introduction

In an aging society, major bone defects caused by trauma, infections, tumors and abnormal skeletal development are a growing therapeutic challenge in orthopedics and trauma surgery^1^. The most common regenerative approach is the use of autologous bone grafts, but this option is often limited by restricted availability as well as substantial donor-site morbidity^2^. Therefore, osteogenic cells such as mesenchymal stromal cells (MSC) and fibroblasts (FB) have been investigated in regenerative bone tissue engineering strategies, and promising results have already been published^3,4^. As demonstrated for adipose-derived stromal cells (ASC), donor site morbidity is decreased, the angiogenic and osteogenic differentiation potential is elevated and the frequency and yield of colony forming units is up to 500-fold enhanced in ASC compared to bone marrow cells^5^. A further interesting cell source are FB because according to the guidelines of the International Society for Cellular Therapy (ISCT), they express specific MSC markers^6^, are plastic-adherent and show multipotent differentiation capacity^7^. FB are, as an essential element of connective tissue, ubiquitously available in the body but are also available in the dermis. FB easily proliferate and have as MSC immunomodulatory and wound-healing features^8–11^. Dental pulp stromal cells (DSCs) in turn are of ectomesenchymal origin^12^, highly proliferative, and precommitted towards hard tissue^13^.

Although FB, ASC, and DSC are multipotent and have osteogenic differentiation potential, they vary widely in their differentiation potential and features^14,15^. One reason could be that MSC with different tissue origins use different modes of action to become osteoblastic cells. In this context, the TGF-β and BMP signaling pathways have a major role because these signaling cascades show crosstalk and are important for osteogenic differentiation since dysregulated signaling results in multiple bone disorders^16^. This phenomenon is underlined by the fact that inconsistent studies describe different effects of BMP-2 and TGF-β, which can induce corresponding pathways. High doses of BMP-2 can accelerate osteogenic differentiation in BMSC or pig ASC^17,18^. In stem cells from individuals with Marfan syndrome, exogenous BMP-2 supplementation antagonized TGF-β signaling and rescued the ability to differentiate osteogenically^19^. But in contrast, BMP-2 did not promote maxillary alveolar reconstruction^20^. Although there is a consensus that endogenous TGF-β^21^ impairs osteoblastic maturation, in C2C12 cells, TGF-β induced the transcription of Runx2, an essential transcription factor required for bone formation^22^.

A further consideration for a safe and optimum therapy in bone tissue engineering strategies is to take into account that for therapeutic use, sufficient cell numbers are required. For a successful in vivo transplantation, at least 1 × 10^6^ cells/kg or 1 × 10^8^ total cells are needed^23^; therefore, some passaging and expansion steps are necessary. But the proliferative capacity and osteogenic differentiation potential of somatic cells decline with cellular senescence^24^. Cellular senescence has serious consequences^25^, probably affecting the osteogenic differentiation potential in FB, ASC and DSC to varying extents. Therefore, we analyzed the effects of TGF-β and BMP signaling in replicative senescence cells. In particular, BMP-2 expression is downregulated in senescent cells^26^, and the osteogenic potential could be restored in aged rats by BMP-2 transduction^27^. In this respect, it is also interesting to note that to date, whether TGF-β itself induces senescence^28^ or not^29^ is unclear.

The aim of the present study was to compare and analyze the effects of replicative senescence on the osteogenic differentiation potential of human MSC with different histological origins, namely, ASC, DSC and FB. Therefore, the impact of TGF-β or BMP-2 signaling, using BMP-2 and specific pathway inhibitors such as SB431542 and dorsomorphin, was evaluated. The objective was to obtain a better understanding of different MSC sources to prospectively improve the safety, adaption and differentiation capacity of transplanted cells in bone tissue engineering strategies.

## Results

### Comparison of the phenotypes of ASC, DSC and FB

**Figure 1:**
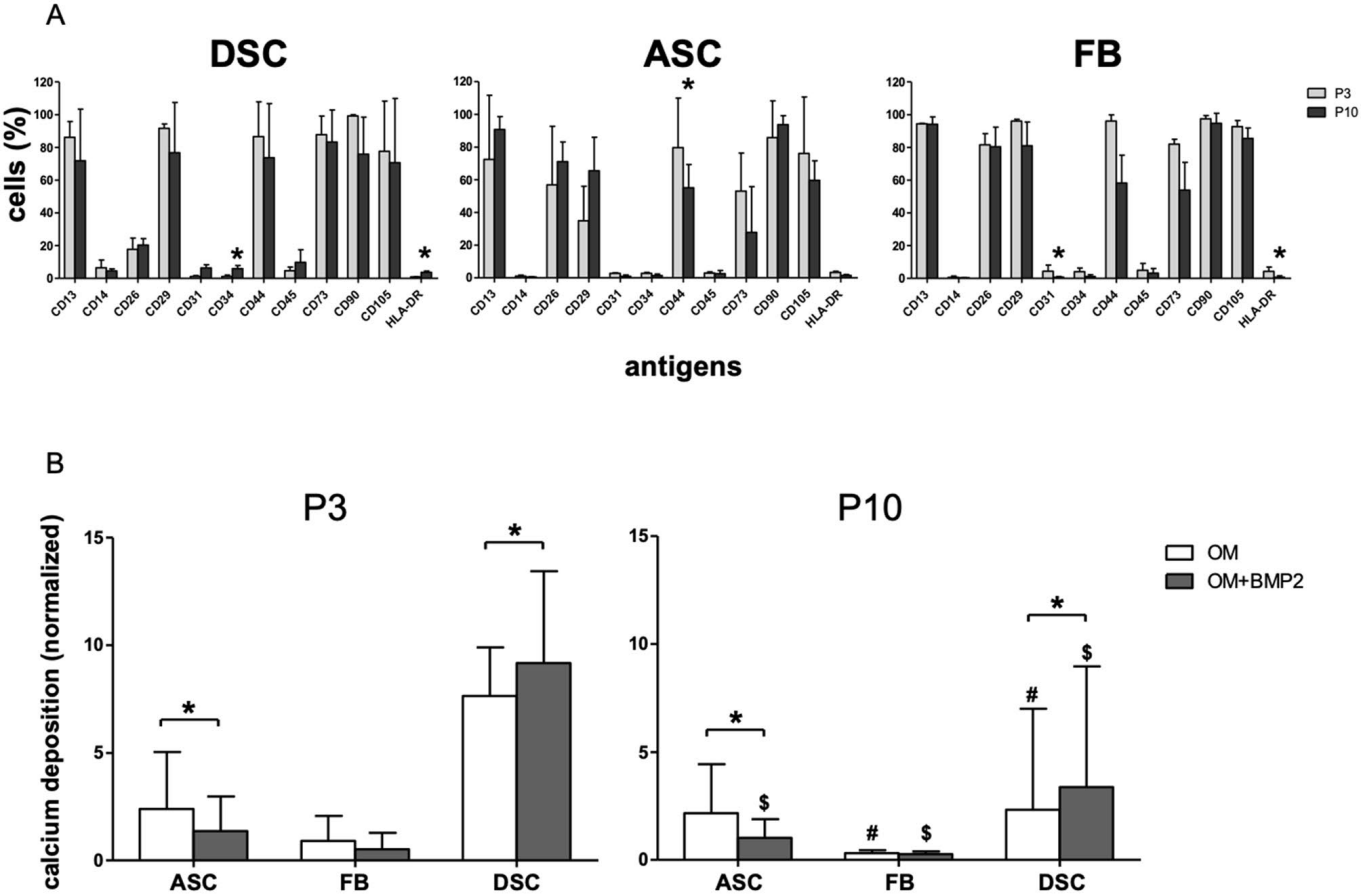
(**A**) Phenotype characterization. Phenotype characterization of human DSC, ASC, and FB cultures in P3 (grey bars) and P10 (black bars) was performed by FACS analysis. Bars represent mean ± SD of three donors. *, *p* < 0.05 as compared to the respective sample of the culture of P10. (**B**) Comparison of the osteogenic differentiation +/− BMP-2 between P3 and P10. The osteogenic differentiation potentials of DSC, ASC, and FB. DSC and ASC at P3 had the best potential to differentiate osteogenically compared to FB. Only in DSC the osteogenic differentiation could be significantly improved with BMP-2. In a comparison of P3 to P10 cells, DSC showed the greatest decrease, and ASC showed the smallest decrease in osteogenic differentiation potential compared to their younger counterparts at P3. White bars demonstrate the osteogenic differentiation with standard osteogenic differentiation media (OM). Grey bars demonstrate the osteogenic differentiation media supplemented with BMP-2 (OM + BMP2). Bars represent mean ± SD of six donors. ^*^p < 0.05 as compared to the respective sample cultured with OM. ^#,$^*p* < 0.05 as compared to the respective sample in P3.

According to the guidelines of the ISCT, ASC, DSC and FB express specific MSC markers at comparable levels. Furthermore, CD29 and CD26 were analyzed for their roles in adhesion and homing interactions^30–32^. Interestingly, CD26 was decreased expressed in DSC compared to ASC and FB. The integrin CD29, which induces cell adhesion^30^, was diminished in ASC compared to FB and DSC (Fig. 1A).

### Effects of senescence

CD44 is a glycoprotein involved in cell–cell interactions, cell adhesion and migration^32^. In a comparison of P3 and P10 cells, decreased expression of CD44 was demonstrated in all analyzed cell types, but this effect was significant only in ASC (Fig. 1A).

### Comparison of the osteogenic differentiation potential of ASC, DSC and FB

**Figure 2:**
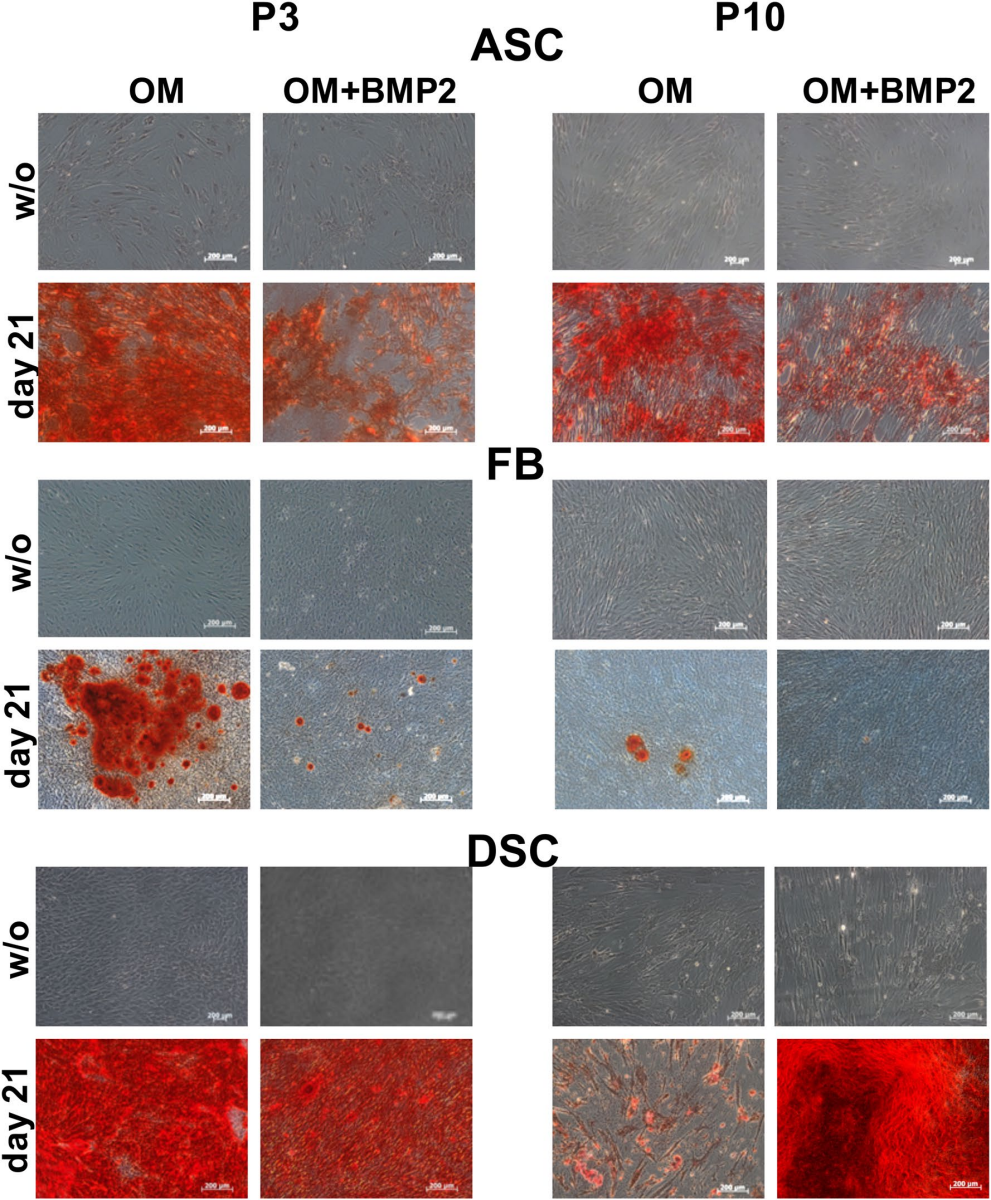
Microscopical analysis of the osteogenic differentiation potential at P3 compared to culture senescence cells at P10. Evaluation was performed with alizarin red s on day 0, and 21. Presented are the untreated controls (without: w/o), cell differentiated with osteogenic differentiation medium (OM) or cells differentiated with OM and supplemented with BMP-2 (OM + BMP2). Shown is one representative illustration of at least six identical results. The image scales represent a length of 200 µm. *p < 0.05 as compared to the respective sample of the culture of P10.

DSC showed the strongest osteogenic differentiation. Moreover, the osteogenic differentiation of DSC could be significantly improved with BMP-2 at P3 and P10. In contrast, in ASC, additional treatment with BMP-2 significantly inhibited the osteogenic differentiation at P3 and P10 (Figs. 1B, 2). In FB, additional treatment with BMP-2 showed almost no difference compared with the standard differentiation media (OM) at P3 and P10.

### Effects of senescence

In a comparison of P3 to P10 cells, ASC showed the smallest decrease in osteogenic differentiation potential, and DSC showed the greatest decrease in osteogenic differentiation potential compared to their younger counterparts (Figs. 1B, 2). The osteogenic differentiation capacity of FB and DSC deteriorated significantly at P10. In ASC, there was no significant difference between the osteogenic differentiation potential (OM) at P3 and P10.

### Expression of high-affinity binding receptors for BMP-2 (ALK-3 and ALK-6) in ASC, DSC and FB

**Figure 3:**
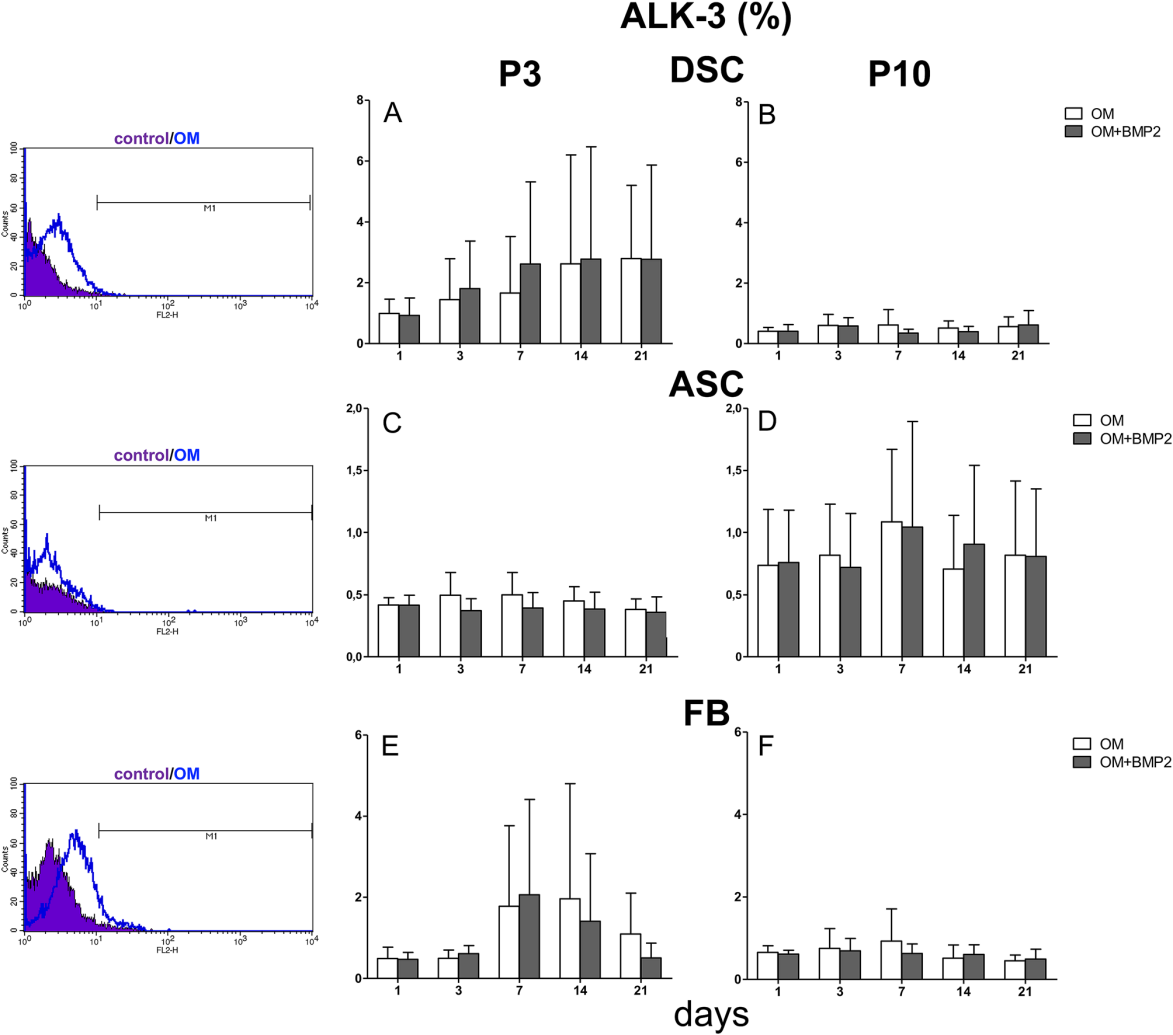
ALK-3 expression in P3 versus culture senescence cells at P10. (**A**) Evaluated was the ALK-3 expression in DSC at P3. Expression of ALK-3 increased over the course of osteogenic differentiation. (**B**) Evaluated was the ALK-3 expression in DSC at P10. The ALK-3 was expressed at a small amount at P10. (**C**) Evaluated was the ALK-3 expression in ASC at P3. (**D**) Evaluated was the ALK-3 expression in ASC at P10. ALK-3 showed a minor expression. (**E**) Evaluated was the ALK-3 expression in FB at P3. The expression increased slightly on day 7 and 14, but decreased again at day 21. (**F**) Evaluated was the ALK-3 expression in FB at P10. Grey bars represent these cells incubated with osteogenic differentiation media supplemented with BMP-2. White bars represent cells differentiated with standard osteogenic differentiation media (OM). Bars represent mean ± SD of three donors.

**Figure 4:**
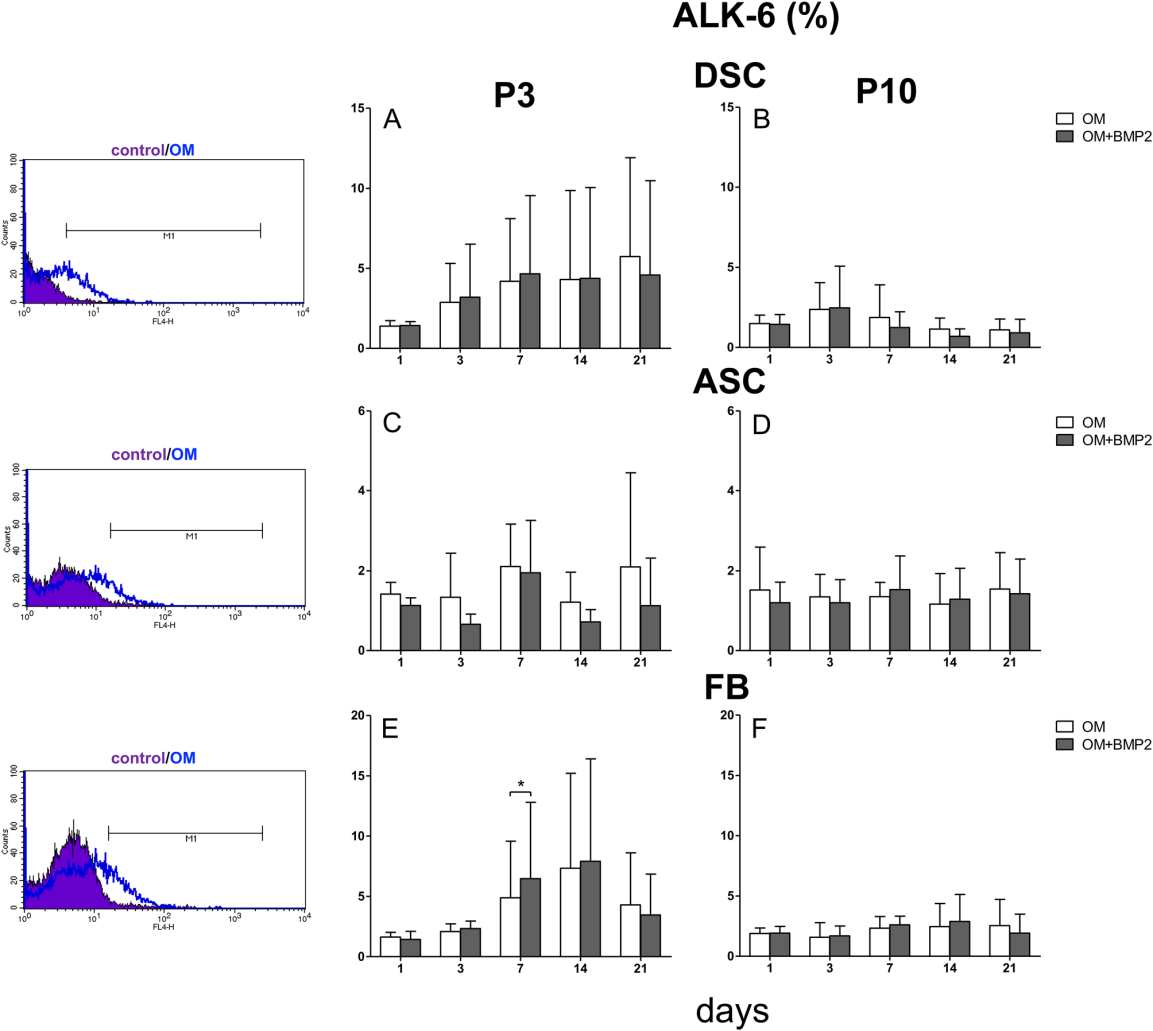
ALK-6 expression in P3 versus culture senescence cells at P10. (**A**) Evaluated was the ALK-6 expression in DSC at P3. Expression of ALK-6 increased over the course of osteogenic differentiation. (**B**) Evaluated was the ALK-6 expression in DSC at P10. (**C**) Evaluated was the ALK-6 expression in ASC at P3. (**D**) Evaluated was the ALK-6 expression in ASC in P10. ALK-6 was expressed at a small amount. (**E**) Evaluated was the ALK-6 expression in FB in P3. The expression increased weakly between day 7 and 14. On day 7 was a significant difference between the BMP-2 treatment and the standard osteogenic differentiation in favor to the BMP-2 supplementation. (**F**) Evaluated was the ALK-6 expression in FB at P10. Grey bars represent these cells incubated with osteogenic differentiation media supplemented with BMP-2 (OM + BMP2). White bars represent cells differentiated with standard osteogenic differentiation media (OM). Bars represent mean ± SD of three donors. *p < 0.05 as compared to the respective sample treated with OM.

In DSC, which were sensitive to BMP-2 supplementation, the expression of the high-affinity binding BMP-2 receptors ALK-3 and ALK-6 increased during osteogenic differentiation at P3 (Figs. 3, 4). Although BMP-2 did not improve the osteogenic differentiation of FB at P3, increased expression of ALK-3 and ALK-6 on days 7 and 14 could be demonstrated, and this effect was significant on day 7 for ALK-6 (Figs. 3, 4).

### Effects of senescence

In general, in DSC and FB, ALK-3 and ALK-6 receptor expression was substantially reduced at P3 compared with P10, similar to the osteogenic differentiation potential. In ASC, both receptors were barely expressed, and little difference was observed between P3 and P10 (Figs. 3, 4), similar to the osteogenic differentiation potential.

### Impact of TGF-β signaling and BMP signaling on osteogenic differentiation

**Figure 5:**
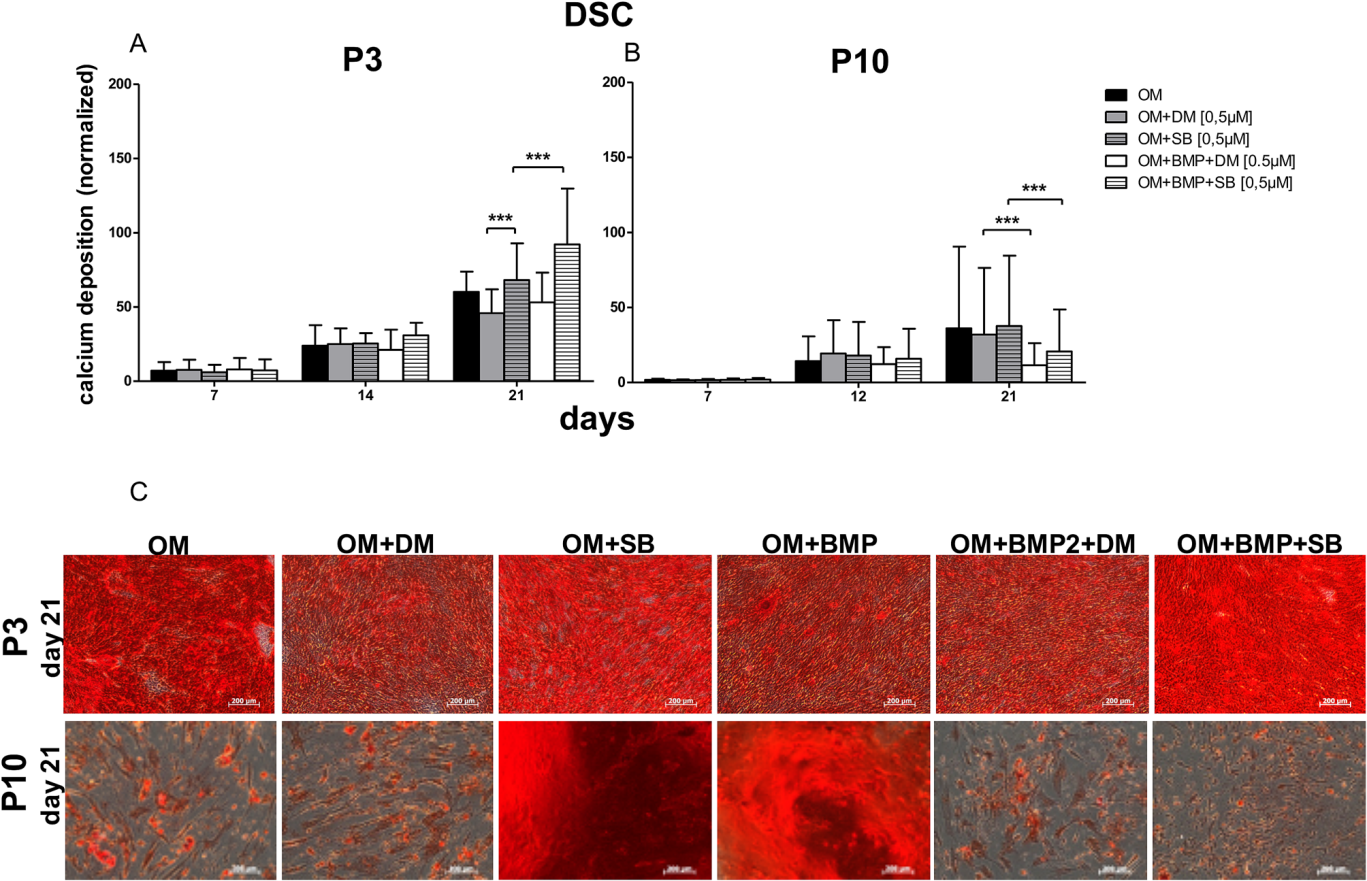
Comparison of the DSC osteogenic differentiation potential at P3 versus P10. (**A**) Evaluation of osteogenic differentiation potential at P3 with alizarin red s. At day 21 the inhibition of TGF-β signaling with SB431542 (OM + SB) was significantly superior to the inhibition of BMP-2 signaling with dorsomorphin (OM + DM). If TGF-β signaling was inhibited with SB431542 and the BMP-2 signaling was induced with BMP-2 (OM + BMP + SB) the osteogenic differentiation was significantly superior to a solely inhibition of TGF-β signaling (OM + SB). (**B**) Evaluation of osteogenic differentiation potential at P10 with alizarin red s. At day 21 the inhibition of TGF-β signaling in the course of osteogenic differentiation with SB431542 (OM + SB) was significantly superior to the inhibition of BMP-2 signaling with dorsomorphin (OM + DM), and superior (in the contrary to P3) to the inhibition of TGF-β signaling with SB431542 and inducing BMP signaling with BMP-2 (OM + BMP + SB). Additionally, the inhibition of the BMP-2 signaling with dorsomorphin (OM + DM) was superior to the osteogenic differentiation with dorsomorphin and BMP-2 (OM + BMP + DM). Black bars represent DSC differentiated with osteogenic standard differentiation media (OM). Grey bars demonstrate DSC differentiated with OM and dorsomorphin (OM + DM). Stacked grey bars are DSC differentiated osteogenically and treated with SB431542 (OM + SB). White bars are DSC coincubated with OM and BMP-2 with the inhibitor dorsomorphin (OM + BMP + DM). Stacked white bars are DSC differentiated with OM, BMP-2 and the inhibitor SB431542 (OM + BMP + SB). Bars represent mean ± SD of three donors. ***p < 0.001 as compared to the respective sample. (**C**) Visualized comparison of osteogenic differentiation potential of DSC at P3, and P10. Evaluation was performed with alizarin red s on day 21. Shown is one representative illustration of at least six identical results. The image scales represent a length of 200 µm.

**Figure 6:**
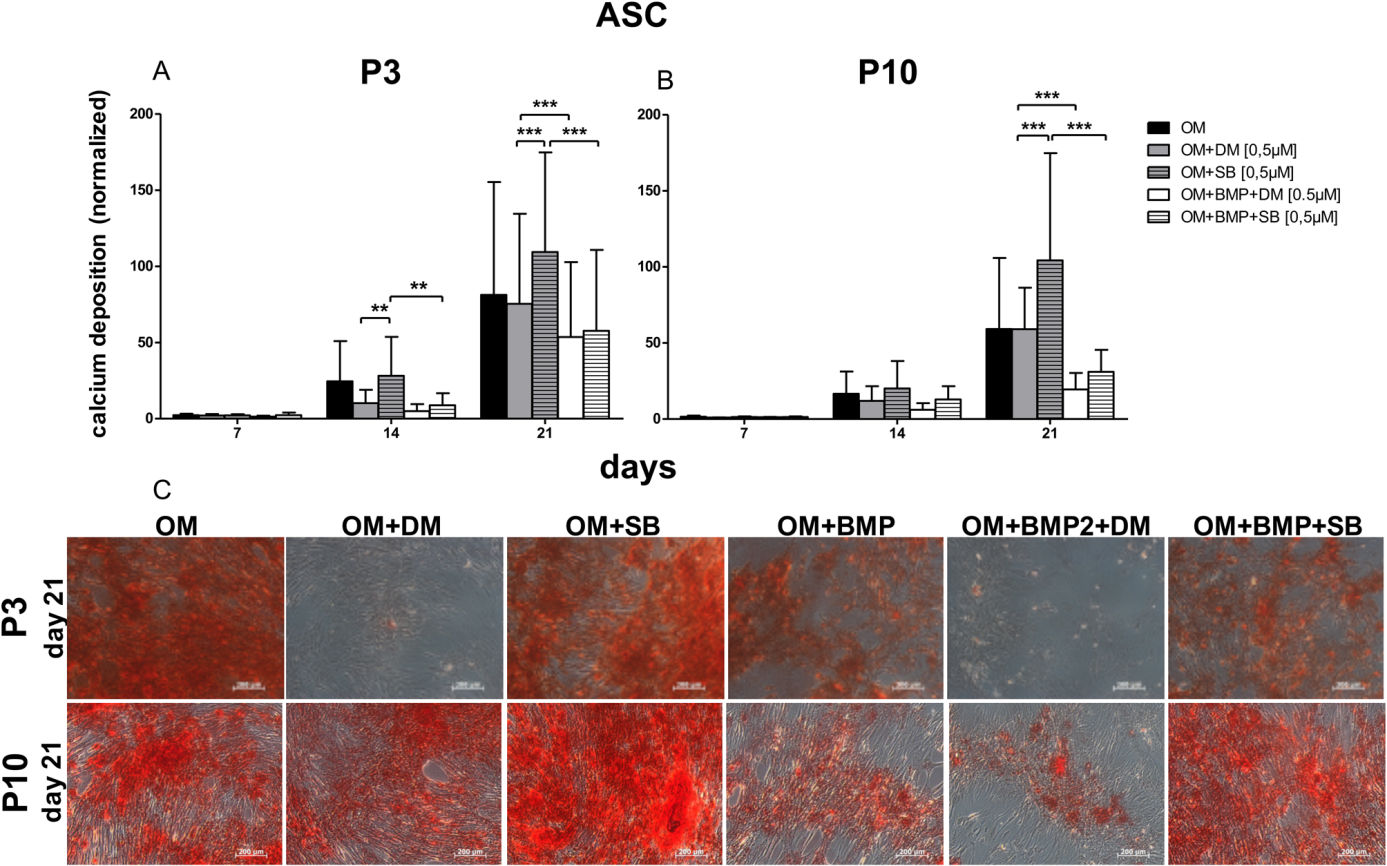
Comparison of the ASC osteogenic differentiation potential at P3 versus P10. (**A**) Evaluation of osteogenic differentiation potential in P3 with alizarin red s. At day 14 the inhibition of the TGF-β signaling with SB431542 (OM + SB) was significantly superior to the inhibition of BMP signaling with dorsomorphin (OM + DM). And the inhibition of the TGF-β signaling significantly was better than the inhibition of TGF-β signaling and the simultaneous induction of BMP signaling with BMP-2 (OM + BMP + SB). (**B**) Evaluation of osteogenic differentiation potential at P10 with alizarin red s. Inhibiting the TGF-β signaling with SB431542 (OM + SB431542) generated the best osteogenic differentiation potential, at day 21 and was significantly superior to an additionally application of BMP-2 (OM + BMP + SB431542). And the inhibition of BMP signaling with dorsomorphin in the course of the osteogenic differentiation (OM + DM) was significantly superior to the treatment with dorsomorphin supplemented with BMP-2 (OM + BMP + DM). Black bars are ASC differentiated with osteogenic standard differentiation media (OM). Grey bars are ASC differentiated with OM and dorsomorphin (OM + DM). Stacked grey bars are ASC differentiated osteogenically and treated with SB431542 (OM + SB). White bars are ASC coincubated with OM and BMP-2 with the inhibitor dorsomorphin (OM + BMP + DM). Stacked white bars are DSC differentiated with OM, BMP-2 and the inhibitor SB431542 (OM + BMP + SB). Bars represent mean ± SD of three donors. **p < 0.01 as compared to the respective sample. ***p < 0.001 as compared to the respective sample. (**C**) Visualized comparison of osteogenic differentiation potential of DSC at P3 and P10. Evaluation was performed with alizarin red s on day 21. Shown is one representative illustration of at least six identical results. The image scales represent a length of 200 µm.

**Figure 7:**
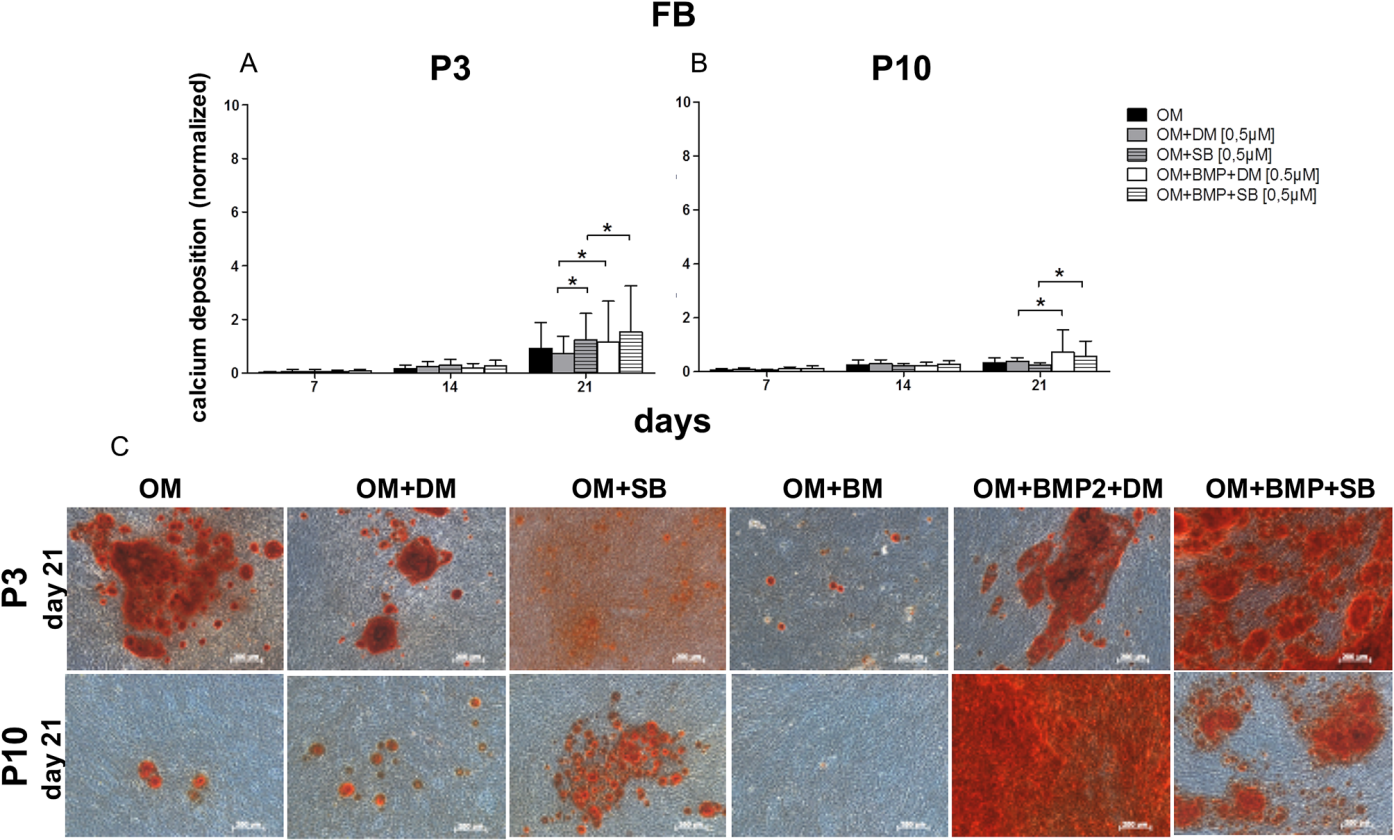
Comparison of the FB osteogenic differentiation potential at P3 versus P10. (**A**) Evaluation of osteogenic differentiation potential at P3 with alizarin red s. The osteogenic differentiation increased over the time course. At day 21 the inhibition of the TGF-β signaling with SB431542 (OM + SB) was significantly superior to the inhibition of BMP signaling with dorsomorphin (OM + DM) and even better than the inhibition of TGF-β signaling with the simultaneous use of BMP-2 (OM + BMP + SB). The differentiation with BMP-2 and simultaneous inhibition of the BMP signaling with dorsomorphin (OM + BMP + DM) was significantly better than the osteogenic differentiation with BMP-2 alone (OM + BMP). (**B**) Evaluation of the osteogenic differentiation potential at P10 with alizarin red s. At day 21 the osteogenic differentiation with BMP-2 with the simultaneous inhibition of the BMP signaling (OM + BMP + DM) was the best and significantly superior to the treatment with dorsomorphin only (OM + DM). Inhibiting the TGF-β signaling with SB431542 and the additional use of BMP-2 (OM + BMP + SB) was significantly superior to the inhibition of TGF-β signaling with SB431542 (OM + SB). Black bars are FB differentiated with osteogenic standard differentiation media (OM). Grey bars are FB differentiated with OM and dorsomorphin (OM + DM). Stacked grey bars were FB differentiated osteogenically and treated with SB431542 (OM + SB). White bars were FB coincubated with OM and BMP-2 with the inhibitor dorsomorphin (OM + BMP + DM). Stacked white bars are FB differentiated with OM, BMP-2 and the inhibitor SB431542 (OM + BMP + SB). Bars represent mean ± SD of three donors. *p < 0.05 as compared to the respective sample of the culture of P10. (**C**) Visualized comparison of osteogenic differentiation potential of DSC at P3 and P10. Evaluation was performed with alizarin red s on day 21. Shown is one representative illustration of at least six identical results. The image scales represent a length of 200 µm.

At P3 of all analyzed cell types, the osteogenic differentiation potential increased over the time course, and the additional inhibition of TGF-β signaling was significantly superior to the inhibition of BMP signaling (Figs. 5, 6, 7). Furthermore, if in DSC and FB at P3, TGF-β signaling was inhibited (with SB431542), and BMP signaling was induced with BMP-2, the osteogenic differentiation potential was significantly superior to that of the cells with inhibition of only TGF-β signaling (Figs. 6, 7). Interestingly, if BMP-2 signaling was inhibited with dorsomorphin in FB and BMP-2 was simultaneously used (OM + BMP + DM), osteogenic differentiation was significantly improved compared to differentiation with dorsomorphin alone (OM + DM).

### Effects of senescence

In contrast to those at P3, in culture senescent DSC (in P10), the inhibition of TGF-β signaling and the simultaneous use of BMP-2 had an inhibitory effect on osteogenic differentiation potential (Fig. 5), similar to the observed effect in ASC.

In ASC at P10 (as in P3), the inhibition of TGF-β signaling was the best method to promote osteogenic differentiation, and this approach was significantly superior to standard osteogenic differentiation with OM. At P3 and P10, the use of BMP-2 for osteogenic differentiation was always inferior to the respective controls (with or without inhibitors) (Fig. 6).

In summary, TGF-β signaling and the exogenous activation of BMP-2 signaling inhibited osteogenic differentiation in ASC.

Interestingly, in FB (P3 and P10), the osteogenic differentiation potential was significantly increased by the simultaneous use of BMP-2 with the BMP signaling inhibitor dorsomorphin compared to treatment with dorsomorphin alone (Fig. 7). Moreover, in FB at P10, the inhibition of BMP signaling with the simultaneous use of BMP-2 was superior to all other applications.

### Evaluation of senescence associated β-galactosidase activity

**Figure 8:**
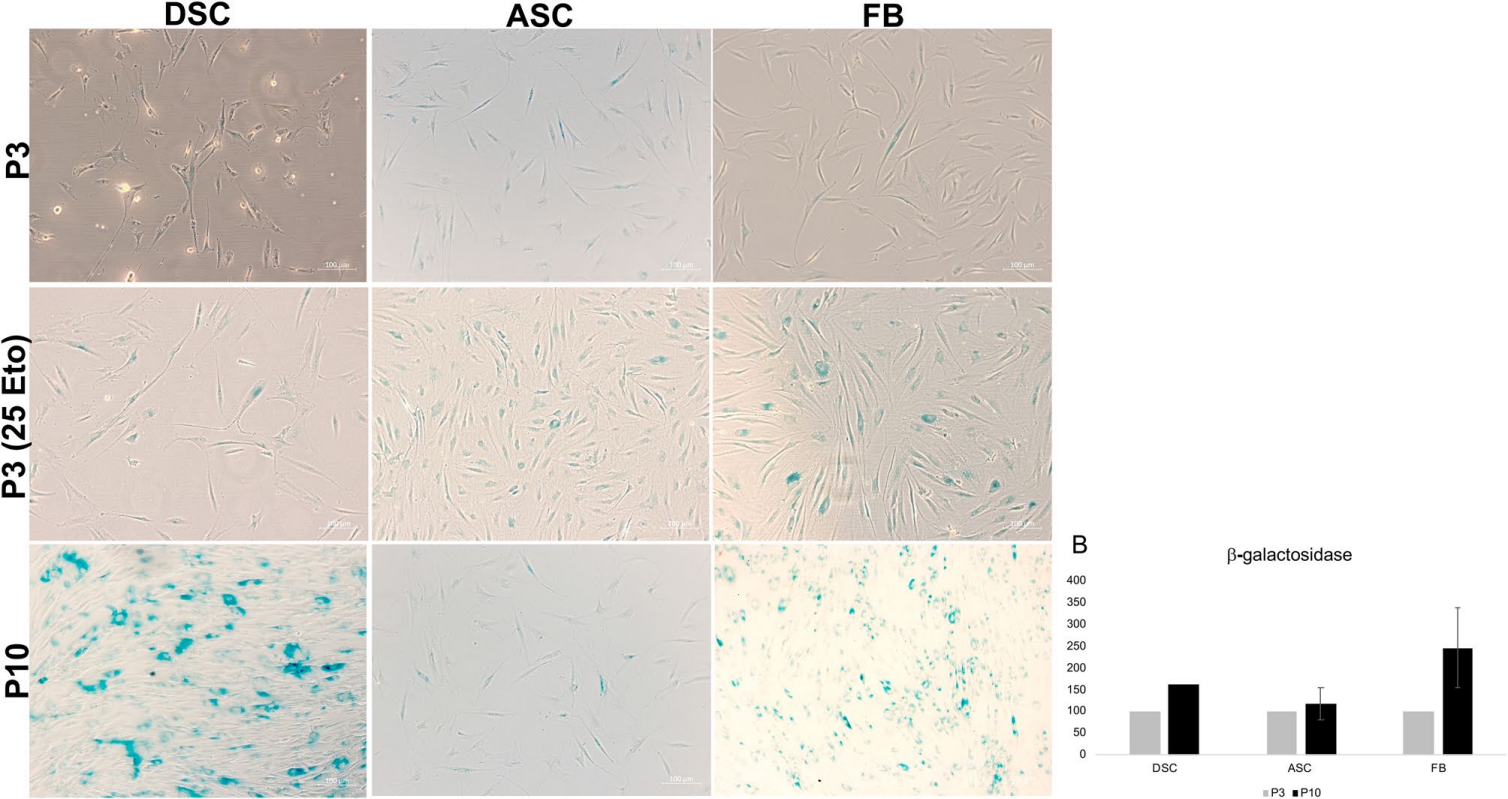
Evaluation of senescence associated β-galactosidase activity. (**A**) Visualized comparison of β-galactosidase activity at P3 and P10**.** At P3 in DSC, ASC and FB β-galactosidase activity was on a comparable level. Control cells treated with etoposide, showed an increase of β-galactosidase activity. At P10 DSC and FB showed a noticeable stronger β-galactosidase staining compared to ASC. Shown are representative illustrations of at least six identical results. The image scales represent a length of 100 µm. (**B**) Evaluation β-galactosidase expression at P3 and P10. In ASC β-galactosidase expression was on a comparable level in P3 and P10. In DSC and FB the β-galactosidase expression noticeably increased in P10 in contrast to P3. Bars represent mean ± SD of two donors.

DSC, ASC and FB at P3 demonstrated a comparable level of β-galactosidase activity visualized in faint blue (Fig. 8). Control cells at P3 treated with etoposide displayed comparable more β-galactosidase.

### Effects of senescence

At P10 DSC and FB exhibited the strongest β-galactosidase activity visualized in blue, in contrast to ASC, which showed a noticeably weaker staining (Fig. 8). Respective β-galactosidase protein expression underlined this observation, that β-galactosidase expression was comparable lower in ASC at P10 in contrast to DSC and FB.

## Discussion

Forty years ago, a pioneering publication by Friedenstein et al*.*^33^ was released describing cells with multipotent differentiation potential. His study suggested that in the near future, destroyed organs or functionless cells could be replaced by MSC. When it was discovered that MSC were available in nearly every tissue, even in the skin, this optimistic mood reached new heights. Today, it is clear that more detailed information is needed about the specific MSC source with regard to their specific applications in regenerative tissue engineering. Moreover, it is known that MSC are precommitted depending on the tissue origin^34,35^ and have varying potential to differentiate osteogenically^36^, but to date, this phenomenon has been insufficiently researched. Compounding the problem is that for an appropriate in vivo application, a high cell number is needed, and therefore, many cell doublings are necessary; this process is inevitably accompanied by replicative senescence and loss of differentiation potential. In this context, TGF-β and BMP signaling has an important role because these signaling cascades regulate bone formation during mammalian development and versatile functions in the body^16^. Against this background, the aim of the current study was to evaluate the impact of TGF-β or BMP signaling on the osteogenic differentiation potential of ASC, DSC and FB, taking replicative senescence into account.

### The osteogenic differentiation of DSC

In our study, DSC showed the required antigen profile as described by Dominici^37^ (Fig. 1A). Furthermore, CD26, an intrinsic membrane glycoprotein, was expressed at lower levels in DSC than in FB and ASC. It has been demonstrated that deletion or inhibition of CD26^38^ increases homing transplantation efficiency^31^.

In this study DSC followed by ASC had the best potential to differentiate osteogenically (Figs. 2, 3). The differentiation potential was significantly improved with BMP-2 supplementation at P3 and P10 (Figs. 1B, 2).

Trivanocic et al*.* showed that DSC are superior in their osteogenic differentiation behavior^39^, and further working groups also observed strong effects of BMP-2 in DSC^18,40^. Another study concluded that BMP-2 treatment can induce the expression of Runx2 but only in DSC^41^. Runx2 is a transcription factor necessary for the early differentiation from MSC to osteochondroprogenitors. Moreover, we supposed that BMP-2 could induce osteoblast mineralization in human DSC through a Wnt autocrine loop, as described by Rawadi et al*.* in various cell lines: C3H10T1/2, C2C12, ST2 and MC3T3-E1^42^. Alternatively, Wnt and BMP signaling may cooperate and synergize to support osteoblast differentiation, as described by Mbalaviele^43^. An indication was our own findings (Suppl 2A) because incubation with BMP-2 led to increased expression of β-catenin, the central factor of canonical Wnt signaling. When Wnt signaling is activated, β-catenin accumulates in the cytoplasm and nucleus, where it can induce target gene expression. And Wnt pathway activity is needed throughout osteogenesis^44^.

While the expression of BMPs in bone development and repair is well documented^45,46^, the associated BMP receptor expression levels have not been elucidated in detail^47^. ALK-3 and ALK-6 are high-affinity binding receptors for BMP-2 and are substantially involved in BMP signaling and osteogenic differentiation in bone^47^. Overall, the elevated osteogenic differentiation capacity in DSC coincided with the increased ALK-3 and ALK-6 expression at P3 (Figs. 3A, 4A).

Consistent with the aforementioned results, a target-oriented inhibitor of BMP signaling, dorsomorphin, significantly reduced the DSC differentiation potential at P3 on day 21 (Fig. 5). BMP-2 signaling interacts with Smad1, Smad5, Smad8 and the common Smad4 to translocate to the nucleus and initiate the expression of osteoblastic genes^48^. Dorsomorphin is a reversible inhibitor that prevents the ligand-associated activation of ALK-2, ALK-3 and ALK-6, which are responsible for the activation of BMP signaling^49^. This signaling can induce the expression of genes indispensable for osteogenic differentiation, such as Runx2 and Osterix^50,51^. Osterix inhibits osteoblast proliferation while inducing osteoblast terminal differentiation^52^. This inhibition is partially mediated through a feedback control mechanism involved in bone formation by decreasing Wnt signaling^53,54^.

Furthermore, the simultaneous use of BMP-2 and the inhibitor of TGF-β signaling, SB431542, significantly improved DSC osteogenic differentiation potential at P3 (Fig. 5). BMP signaling and TGF-β signaling can crosstalk and compete for executor Smad4^55^. In TGF-β signaling, Smad2 and Smad3 are phosphorylated, interact with the common Smad4 and translocate to the nucleus together, where they recruit further cofactors to regulate gene transcription. SB431542 selectively inhibits the kinase activity of the TGF-β receptors ALK-4, ALK-5 and ALK-7, and thus, Smad2 and Smad3 could not be activated by TGF-β or activin. Therefore, the use of BMP-2 with the simultaneous inhibition of TGF-β-signaling most likely led to an acceleration of BMP signaling, as described by Maeda et al*.*^21^.

### Osteogenic differentiation in DSC with replicative senescence

However, in a comparison of P3 to P10 cells, DSC showed the greatest decrease, and ASC showed the smallest decrease in osteogenic differentiation potential compared to their younger counterparts at P3 (Fig. 1B). This observation could be underpinned with senescence-associated β-galactosidase staining at P10, because in DSC β-galactosidase activity was stronger as in ASC (Fig. 8). The significantly decreased capacity for differentiation was accompanied by decreased ALK-3 and ALK-6 expression at P10. Additionally, the inhibition of TGF-β signaling did not improve osteogenic differentiation potential in DSC at P10. Similar results were described by Patel et al., who observed that at P10 in DSC, some genes related to osteogenic differentiation were clearly downregulated^56^. Furthermore, this finding could indicate that TGF-β is probably not responsible for replicative senescence in DSC, as was declared by Walenda et al*.*^29^.

### The osteogenic differentiation of ASC

CD29, which induces cell adhesion^30^, showed decreased expression in ASC compared to FB and DSC (Fig. 1A), but antigen expression of the factors described by Dominici was as expected.

Furthermore, the ASC osteogenic differentiation potential at P3 was lower than that of DSCs (Fig. 1B). However, it must be considered that in general, abdominal fat is usually provided by individuals undergoing liposuction or abdominal plastic surgery who are in their forties and fifties^57^, whereas DSCs are donated by younger adults in their twenties when their wisdom teeth are removed^13^. It is commonly accepted that the differentiation potential of MSC decreases with donor age^58^. However, the results published by D’Alimonte et al*.* attested that ASC have a better osteogenic differentiation potential than DSC, but in this study, the donor age difference between DSC and ASC was only ten years (from 18 to 28 years), and therefore, both groups were relatively young^59^.

Compared to that in DSC, BMP-2 supplementation in ASC significantly inhibited their osteogenic differentiation potential at P3 and P10 (Fig. 1B). Alonso et al*.* ^20^ and Dickinson et al.^60^ also demonstrated varying in vivo results with BMP-2. Alonso et al*.* reported lower bone regeneration with BMP-2, and Dickenson et al. demonstrated improved healing in alveolar reconstruction. Another study showed that BMP-2 application is cell-type specific, as BMP-2 did not accelerate osteogenic differentiation potential in mouse fibroblasts but did in myoblasts (C2C12) and in preosteoblasts (MC3T3-E1)^61^.

Consistent with our results, the expression of the BMP-2 receptors ALK-3 and ALK-6 was very low in ASC (Fig. 3C,D and 4C,D). However, interestingly, the application of dorsomorphin to ASCs during osteogenic differentiation had a short-term inhibitory effect on day 14 at P3 (Fig. 6). This finding emphasizes that (endogenous) BMP signaling is important in the early phase of osteogenic differentiation in ASC, but as described by Zuk et al*.*^62^, it plays a subordinate role. In ASC the inhibition of TGF-β signaling significantly accelerated the osteogenic differentiation potential to similar levels at P3 and P10 (Fig. 6). These findings are supported by the fact that SB431542 induces osteogenic differentiation in C2C12 cells and promotes matrix mineralization^21^. We assume that in ASC, osteogenic differentiation is mediated by phosphorylating TAK1 and TAB1 by inducing the MAP kinase pathway (p38 and MAPK-ERK1/2) rather than by BMP signaling. Our assumption was supported by a protein analysis, which exemplary showed elevated p38 expression in ASC treated with SB431542 (Suppl 2B). MAPK signaling can also promote osteoblastic master transcription factors, such as Runx2 and Osterix^55^.

### Osteogenic differentiation in ASC with replicative senescence

In a comparison of P3 and P10 cells, decreased expression of CD44 was demonstrated in all analyzed cell types, but this effect was significant only in ASC (Fig. 1A). CD44 is a glycoprotein involved in cell–cell interactions, cell adhesion and migration^32^.

The ASC osteogenic differentiation potential at P10 barely decreased compared to that at P3 (Fig. 1B) and the osteogenic differentiation pattern did not change between P3 and P10. Therefore, BMP-2 supplementation significantly inhibited osteogenic differentiation, whereas the inhibition of TGF-β signaling significantly accelerated the osteogenic differentiation potential (Fig. 6).

Additionally, a study by Beane et al*.*^58^ similarly determined that ASC from older patients were not as affected by senescence as bone marrow- or muscle-derived stromal cells. Another working group evaluated, that in vitro and in vivo properties in ASC were mostly maintained during aging^63^. Consistent with these findings, we demonstrated that β-galactosidase activity, a known characteristic of senescent cells, in ASC was expressed at a comparable level at P3 and P10 (Fig. 8). Furthermore, in ASC at P10, Osterix protein expression was highly expressed even in untreated cells (Suppl 1B).

### The osteogenic differentiation of FB

FB expressed the antigens described by Dominici^37^ (Fig. 1A). The osteogenic differentiation potential of FB was comparatively low at P3 and even worse at P10. Delayed Osterix protein expression could jointly be responsible for the decreased differentiation potential of FB at P3 and P10 because Osterix expression increased only on day 14, indicating that FB needed more time for osteogenic differentiation (Suppl 1C).

Although the application of BMP-2 did not improve differentiation (Figs. 1B, 2), the simultaneous use of BMP-2 with the parallel inhibition of TGF-β was significantly superior to the sole inhibition of TGF-β-signaling at P3 and P10 (OM + BMP + SB; Fig. 7).

Furthermore, the following observations were made at P3 and P10: if BMP-2 was used and BMP-2 signaling was simultaneously inhibited with dorsomorphin (OM + BMP + DM), osteogenic differentiation was significantly improved compared to differentiation with dorsomorphin alone (OM + DM). Based on these results, we concluded that BMP signaling plays a substantial role in human FB, especially considering that the expression of the BMP receptors ALK-3 and ALK-6 was increased during osteogenic differentiation at P3 and that ALK-6 expression was significantly elevated due to the additional BMP-2 treatment on day 7 (Figs. 3E, 4E).

However, perhaps the applied BMP-2 concentration in FB at P3 was too high and initiated a feedback loop because the effect of BMP-2 is dependent on its concentration^18^, the same concentrations were used in all analyzed cell types in our study for better comparability. This assumption was underlined by the fact that SB431542, a TGF-β signaling inhibitor, reduces the nuclear accumulation of Smads^64^. This finding could explain why the simultaneous usage of BMP-2 and SB431542 could accelerate the osteogenic differentiation potential compared to BMP-2 application only.

Moreover, BMP-2 and TGF-β signaling have time-dependent preferences. BMP-2 signaling via Smad1, Smad5 and Smad8 supports early osteogenic differentiation in particular and late osteogenic differentiation. TGF-β signaling induces osteogenic differentiation in the early stages but inhibits osteogenic differentiation in the later phases^55^.

As expected and consistent with the aforementioned results in ASC and DSC the inhibition of TGF-β signaling (OM + SB) was significantly more beneficial than the inhibition of BMP-2-signaling at P3 (Fig. 7).

### Osteogenic differentiation in FB with replicative senescence

Osteogenic differentiation declined significantly between P3 and P10. In FB at P10, ALK-3 and ALK-6 expression was significantly reduced, as was the capacity for osteogenic differentiation (Figs. 3B, 4B). In line with this in FB at P10 β-galactosidase protein expression as a characteristic for senescence was the highest in contrast to ASC and DSC (Fig. 8). Nevertheless, the simultaneous use of BMP-2 and the inhibition of BMP signaling tended to be the best choice at P10 (Fig. 7).

Our study has several limitations. For our study, we preferred to use primary cells, although these cells show interpersonal donor variabilities^65^. In our opinion, this approach was closer to a prospective clinical application. Although donor age affects differentiation potential^66^, we were not allowed to document the patient’s age because of the limitations of our ethical approval. However, even MSC from young donors demonstrated differences in their differentiation potential and clinical usefulness^67^, and to extrapolate this fact, MSC from the same donor with the same tissue origin obtained over a six-month period exhibited differences^67^.

In our work, the cell number of individual donors was extended as much as possible to monitor donor-specific differences. Therefore, we found in individual cases that a good osteogenic differentiation performance correlated, e. g., with high p38 expression in ASC treated with SB. However, this phenomenon was accompanied by a lack of significance in the Western blot analysis. The lack of significance was also because osteogenic differentiation is a gradual process, and most involved proteins have peak time of expression.

Furthermore, for better comparability, we used the same BMP-2, dorsomorphin and SB concentrations in ASC, DSC and FB according to a cell viability assay (Suppl 3A–F). Therefore, it might be possible that the applied concentrations were not the optimal concentrations for every cell type.

TGF-β signaling plays a decisive role in osteogenic differentiation, but beyond that, it is presumed that TGF-β activates cellular senescence by inducing the p16 and p21 pathways^68^. Additionally, Kawamura et al*.*^28^ reported that TGF-β2 is one of the candidate genes for aging in double-positive mesenchymal stromal cells (DPMSC), particularly because old DPMSCs contain significantly more TGF-β2. And the observed aging phenomena were reversed in old DPMSC using a TGF-β antibody (1D11). Interestingly, our observations regarding this matter indicate that TGF-β signaling is not responsible for replicative senescence, although we did not use SB431542 over the course of passaging from P0 to P10. However, for a proper analysis concerning TGF-β-signaling and SB431542 in the context of senescence (which was not our objective), altered pre-mRNA processing, ROS content, disturbed proteostasis, increased mitobiogenesis, etc. should be evaluated.

In contrast, our study has many strengths, it could be demonstrated that DSC have the best osteogenic differentiation potential at P3. DSC differentiate osteogenically using SMAD-dependent BMP-2 signaling, which coincides with elevated ALK-2 and ALK-3 expression. Consistent with this finding, the differentiation potential can be further accelerated with BMP-2. However, as a source for cell-based therapies DSC are likely improbable because potential patients often have their wisdom teeth removed, and an additional growth factor treatment to boost osteogenic differentiation of DSC is critically assessed. Furthermore, an appropriate cell number is required to treat large bone defects, and in DSC, this is accompanied by replicative senescence and impaired differentiation potential. A similar effect was observed by Mehrazarin et al*.* with stromal cells derived from different dental tissues^69^. Therefore, DSC are suggested for the treatment of immune disorders such as graft versus host disease because of their potential homing capabilities or for the treatment of minor defects in endodontics^70,71^.

ASC use in particular the MAP kinase pathway to differentiate osteogenically. From our presented data, it appears that ASC are more genetically stable, have a greater senescence ratio, and retain their differentiation potential for a longer period in cell culture. BMP-2 is not needed to accelerate ASC osteogenic differentiation potential. Thus, ASCs are favorable candidates for bone tissue engineering strategies. ASC therapeutic usefulness has already been demonstrated in wide-ranging clinical applications, such as wound healing^72^, type 1 diabetes mellitus^73^, osteoarthritis^74^, autoinflammatory diseases^75^, age-related macular degeneration and Stargardt’s macular dystrophy^76^.

SMAD-dependent BMP signaling plays a functional role in FB osteogenesis, but the osteogenic differentiation potential is comparably lower and declines with replicative senescence. Either the optimal differentiation protocol has not yet been evaluated or possibly the gradual process of FB osteogenic differentiation takes much longer, as suggested by delayed Osterix expression. Nevertheless, initial and promising approaches were made to evaluate FB therapeutic potential. Therefore, gingival fibroblasts have been used for the treatment of periodontal intrabony defects^77^, and a multicenter study confirmed the usefulness of FB in treating chronic foot ulcers^78^.

These insights could provide important information for the target-oriented use of ASC, DSC and FB in bone tissue engineering strategies. In conclusion, it could be demonstrated that every cell type prefers different signaling cascades to become osteoblastic cells. Furthermore, the analyzed DSC, ASC and FB vary in their potential to differentiate osteogenically depending on their tissue origin and replicative senescence. This is also an important finding; to date, researchers have used and compared various cell types at different cell culture passages in their in vitro and in vivo experiments, and according to our results, this is not a proper approach.

## Materials and methods

### Donors

Due to ethical approval, it was not possible to note donor age, sex, race, body mass index or donor diseases. According to the experiences of other working groups, we assume that the DSC donors are mostly approximately twenty years old^13,79^. FB and ASC isolated from abdominal plastic surgery are usually donated by middle-aged women in their forties and fifties^57,80^. The DSC tissue was derived from impacted molars, whereas ASC and FB were mostly isolated from abdominal plastic surgery, and a rarer event was isolation from thighs. The study design was as follows: DSCs, ASCs and FBs were isolated from three donors, and the starting cell material was spread to the maximum and used for the osteogenic differentiation analysis at P3 and P10. These DSC, ASC and FB donors were used for experiments considering osteogenic differentiation ± BMP-2, treatments with respective inhibitors and Western blot analysis. For phenotypic characterization, further donors were evaluated, and for cell viability and senescence assays, another donor was evaluated. The cells were not pooled to prevent cells from one donor from overgrowing another in the course of passaging.

### Materials

Unless specified otherwise, all chemicals and cell culture materials were obtained from Merck KGaA, Darmstadt, Germany.

### Isolation and culture of FB

Briefly, in accordance with an established protocol^7^, skin samples from abdominoplasty were cut into 5 mm^2^ small pieces and treated for 12 h at 4 °C with dispase II-solution (0.2%) (Roche Diagnostics, Mannheim, Germany) to separate the dermis from the epidermis. To separate human skin fibroblasts, we digested the obtained dermis tissue with 0.2% collagenase I (type: CLS 255 U/mg) and 1.5% BSA in collagenase buffer (100 mM HEPES, 120 mM NaCl, 50 mM KCl, 1 mM CaCl_2_, 5 mM glucose in aqua dest.) for 45 min at 37 °C in a shaking water bath. Dermal remnants were removed by filtering the digest through a 100 mm nylon strainer (Falcon, Becton Dickinson [BD], San Jose, USA). The fibroblasts were subsequently washed with PBS and expanded in standard cultivation medium.

### Isolation and culture of ASC

ASC were isolated from freshly excised human subcutaneous abdominal adipose tissue^81^. Adipose tissue was cut into small pieces (5 mm^2^) and digested with collagenase solution type I (type: CLS 255 U/mg) (0.2%) at 37 °C for 45 min with constant shaking. The ratio for tissue to enzyme was 1:1. After filtration (100 µm), the fat layer was removed, and the cell suspension was centrifuged at 300×*g* for 7 min. After resuspension, the cells were seeded in cell culture flasks and cultured in standard cell culture medium.

### Isolation and culture of DSC

After the impacted molars were broken, the pulp obtained was digested with collagenase solution type I (0.2%) at 37 °C for 45 min with constant shaking^82^. After digestion, the cell suspension was collected, diluted with phosphate buffered saline (PBS) and centrifuged at 300×*g* for 10 min. The pellet was suspended, and the cells were seeded in cell culture flasks_._

### Standard cell cultivation

DSC, ASC and FB were cultivated in DMEM (4.5 g/L glucose) supplemented with 2 mM α-glutamine (PAA), 100 U/ml penicillin, 100 µg/ml streptomycin (PAA) and 10% fetal bovine serum (FBS) (Biochrom, Berlin, Germany). Before reaching subconfluence, the cells were split at a ratio of 1:3. The cells were cultured in 75 cm^2^ culture flasks or seeded in 6- or 24-well plates for osteogenic differentiation and maintained at 37 °C in a humidified atmosphere containing 5% CO_2_.

### Osteogenic differentiation with or without BMP-2

Osteogenic differentiation was induced with osteogenic differentiation medium (OM) based on standard cultivation medium containing 50 µM α-ascorbate-2-phosphate, 10 mM β-glycerophosphate, and 0.1 µM dexamethasone^83^. Medium was replaced twice a week. Alternatively, OM was supplemented with 450 ng/ml BMP-2 (PeproTech, Hamburg, Germany)^17^. After 0, 7, 14 and 21 days, the osteogenic differentiation capacity was determined using the alizarin red S assay as described below. For each time point, a sextuple determination was performed, and the value at day 0 was subtracted.

### Inhibition of TGF-β and BMP-2 signaling with dorsomorphin and SB431542

For inhibition of TGF-β and BMP-2 signaling, dorsomorphin and SB431542 were used. The optimal inhibitor concentrations were evaluated using osteogenic differentiation media (OM), and the appropriate inhibitor was added in ascending concentrations. Both dorsomorphin and SB431542 were used at a concentration of 0.5 µM because these concentrations did not significantly affect the cell viability (measured with CellTiter-Blue) of FB, DSC, and ASC over the observation period of 14 days.

### Osteogenic differentiation with BMP-2, dorsomorphin, and SB431542

DSC, ASC and FB were differentiated osteogenically and additionally treated with/without 450 ng/m BMP-2, 0.5 µM dorsomorphin, and 0.5 µM SB431542 (as described above). On days 0, 7, 14, and 21, alizarin red S staining was performed. For each time point, a sextuple determination was performed. The values obtained were normalized to that of day 0, which was mathematically considered “1”.

### Alizarin red s staining

Alizarin red S is a dye that binds selectively to calcium salts and is widely used for calcium mineral histochemistry^84^. Adherent cell monolayers cultured in 6-well plates were washed with PBS and fixed with 4% paraformaldehyde for 15 min, rinsed 2 times with PBS, covered for 20 min at 37 °C with alizarin red S (0.5% in aqua dest., pH 4.1) and washed with dH_2_O until the supernatant was colorless. Stained monolayers were visualized by phase microscopy using an inverted microscope (Zeiss Axiovert 200 microscope). This process was followed by a quantitative destaining procedure using 10% (w/v) cetylpyridinium chloride in 10 mM sodium phosphate, pH 7.0, for 25 min at room temperature. The alizarin red S concentration was determined by absorbance measurement at 600 nm^85^. For each time point, a sextuple determination was performed.

### Phenotypic characterization

The antigenic phenotype of ASC, DSC and FB was characterized using flow cytometry. Cells were detached with 0.5% trypsin and 0.02% EDTA. After a reconstitution step on ice for 15 min, the cells were washed and centrifuged at 300 g for 5 min. Staining was performed with conjugated antibodies against CD73-PE (550257), CD105-APC (562408), CD34-FITC (345801), and HLA-DR-PE (340689) from BD Bioscience; and CD90-PE (45–0909) (eBioscience), CD45-FITC (21270453) and CD14-FITC (21279143) from ImmunoTools, representing the minimal criteria of mesenchymal stromal cells defined by the International Society of Cellular Therapy (ISCT) ^37^. Additionally, the following antibodies were used: CD44-APC (559942; BD Bioscience), CD26-APC (Ma1-10158; Thermo Scientific), CD13-PE (21270134; ImmunoTools), CD29-PE (2170294 ImmunoTools), ALK-3-PE (FAB346P; R&D Systems) and ALK-6-APC (FAB5051A; R&D Systems). According to the manufacturer’s specifications, appropriate staining concentrations were used. Before cells were stained on ice in the dark for 30 min, cells were blocked for 5 min in FBS. The cells were washed with cell washing solution (3% FBS), resuspended in this solution (3% FBS) and analyzed using a FACSCalibur system (BD Biosciences). All experiments included negative controls without antibodies and respective isotype controls. Therefore, mouse IgG1-FITC (555748), mouse IgG2b-APC (555745), mouse IgG2a-APC (562748), mouse IgG1-APC (555751) from BD Bioscience, goat IgG (AB-108-C) from R&D Systems and mouse IgG1-PE (2815014; Immuntools) and mouse IgG2a-PE (400211; from BioLegend) were used.

### β-Galactosidase assay

Cells were seeded in 1.9 cm^2^/well and cytochemically stained at pH 6.0 for senescence-associated β**-**galactosidase with a Senescence-β**-**Gal Staining Kit (Cell Signaling Technology, Massachusetts, USA) according to the manufacturer’s instructions. As a positive control, the cells were treated with etoposide (25 µM, 48 h) and allowed to recover. Respective microscopically images were performed with Zeiss Axiovert 200. Furthermore, Western blot analysis for detection of β**-**galactosidase were performed (description further below). In short proteins at P3 and P10 from DSC, ASC and FB were collected, 40 µg/lane were applicated and incubated with α-β-galactosidase antibody (abcam ab616, 1:2000) and respective protein expression was normalized on total protein^86^.

### Cell viability test (metabolic activity)

The cell number was calculated by using CellTiter-Blue (Promega, Madison, USA). CellTiter-Blue’s working dilution was 1:20 in medium. CellTiter-Blue uses an indicator dye to measure the metabolic activity of cells as indirect evidence for cell viability. After 1 h, the fluorescence (540_Ex_/590_Em_) was measured in a 1420 Multilabel Counter (Victor^3^, Perkin Elmer).

### Western blot analysis

For determination of the protein expression of Osterix, BMP-2, β-catenin, p38 and GAPDH, Western blot analysis was performed. Protein concentration was analyzed with the Pierce BCA Protein Assay Kit (Thermo Fisher). Twenty micrograms of protein was mixed with 5 µl of Laemmli buffer (4 × Tris glycine-SDS sample buffer, 252 mmol Tris–HCl pH 6.8; 40% glycerine; 8% SDS; 0.01% bromophenol blue + 20% mercaptoethanol), centrifuged (12,000 rpm, 5 min at 4 °C), denatured for 5 min at 95 °C, and separated on a 12% sodium dodecyl sulfate–polyacrylamide gel (SDS-PAGE). Separated proteins were transferred with BioRad Trans-Blot Turbo to a nitrocellulose membrane. Then, the membranes were saturated with different antibodies (β-catenin: Abcam ab16051, 1:4,000; BMP-2: R&D Systems MAB3551, 1:4,000; p38: Cell Signaling Cat. No #9212, 1:4,000; Osterix: Santa Cruz Biotechnology Sc-22538 1:4,000; GAPDH Novus Biologicals NBP2-27103, 1:10,000). The antibodies were incubated at 4 °C overnight. Anti-rabbit or anti-mouse conjugated with horseradish peroxidase (HRP) served (1:1,000) as the secondary antibody with 0.025% anti-Western marker in TBS-T, which was added for 1 h (RT). Before and after the addition of the secondary antibody, the membranes were washed three times with TBS-T. Western blots were visualized with Image Lab version 6.0.1 build 34, 2017, Standard Edition, Bio-Rad Laboratories.

### Statistical analysis

Values represent the mean ± standard deviations (SD). Statistical analysis was performed using two-way ANOVA followed by an appropriate post hoc Bonferroni test. Furthermore, we used two-sided Student’s paired *t-*test. A *p* < 0.05 was considered significant.

### Ethics approval and consent to participate

Study approval was obtained from the Ethics Review Board of the Medical Faculty, Heinrich Heine University Düsseldorf (Study No. 3634). All patient-related data were anonymized before analysis. The usage of human material was conducted in compliance with the Declaration of Helsinki Principles. Written informed consent was obtained from all patients. All donors were analysed separately and not pooled, the specific numbers used are indicated in figure captions.

## Supplementary Information

Supplementary Information.

## Data Availability

The data that support the findings of this study are available from the corresponding author on reasonable request.

## Abbreviations

MSC: Mesenchymal stromal cells
BMSC: Bone marrow derived stromal cells
ASC: Adipose derived stromal cells
FB: Fibroblasts
DSC: Dental pulp stromal cells
BMP-2: Bone morphogenic protein-2
TGF-β: Transforming growth factor-β
TAK1: Transforming growth factor-β-activated kinase
TAB1: Transforming growth factor-β-activated kinase binding protein 1
MAP: Mitogen activated protein
p38: Mitogen activated protein kinase 14
ERK: Extracellular signal-regulated kinase
Runx2: Runt-related transcription factor 2
Dlx5: Distal less homebox 5
Smad: Similar to mothers against decapentaplegic
ALK: Activin receptor-like kinase
P3: 3, -6, 7, Cell culture passage 3
P10: Cell culture passage 10
OM: Cell differentiated with osteogenic differentiation media
OM + SB: Cell differentiated with osteogenic differentiation media + SB431542
OM + DM: Cell differentiated with osteogenic differentiation media + dorsomorphin
OM + BMP2: Cell differentiated with osteogenic differentiation media + BMP-2
OM + SB + BMP: Cell differentiated with osteogenic differentiation media + SB431542 + BMP2
OM + DM + BMP: Cell differentiated with osteogenic differentiation media + dorsomorphin + BMP2
