# Author Correction: Osteogenic differentiation of human mesenchymal stromal cells and fibroblasts differs depending on tissue origin and replicative senescence

**DOI:** 10.1038/s41598-021-94497-7

**Published:** 2021-07-27

**Authors:** Vera Grotheer, Nadine Skrynecki, Lisa Oezel, Joachim Windolf, Jan Grassmann

**Affiliations:** Clinic for Orthopedics and Trauma Surgery, Medical Faculty of the Heinrich Heine University, Moorenstr. 5, 40225 Düsseldorf, Germany

Correction to: *Scientific Reports* https://doi.org/10.1038/s41598-021-91501-y, published online 07 June 2021

The original version of this Article contained errors.

Christopher V. Suschek was incorrectly listed as an author of the original Article, and has subsequently been removed.

The Author Contributions section now reads:

V.G. wrote the manuscript, designed and directed the study. N.S. carried out the most experiments. L.O. and J.G. contributed to the sample preparations. J.W. supervised the project. J.G. had substantively revised the manuscript and has helped to interpret the data. All authors provided critical feedback and helped shape the research and manuscript.

In addition, Figure 1 did not display correctly. The original Figure 1 and accompanying legend appear below.

**Figure 1:**
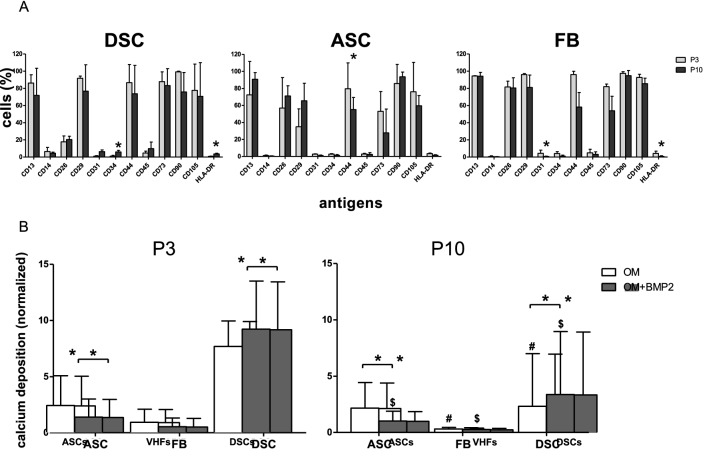
(**A**) Phenotype characterization. Phenotype characterization of human DSC, ASC, and FB cultures in P3 (grey bars) and P10 (black bars) was performed by FACS analysis. Bars represent mean ± SD of three donors. *, *p* < 0.05 as compared to the respective sample of the culture of P10. (**B**) Comparison of the osteogenic differentiation +/− BMP-2 between P3 and P10. The osteogenic differentiation potentials of DSC, ASC, and FB. DSC and ASC at P3 had the best potential to differentiate osteogenically compared to FB. Only in DSC the osteogenic differentiation could be significantly improved with BMP-2. In a comparison of P3 to P10 cells, DSC showed the greatest decrease, and ASC showed the smallest decrease in osteogenic differentiation potential compared to their younger counterparts at P3. White bars demonstrate the osteogenic differentiation with standard osteogenic differentiation media (OM). Grey bars demonstrate the osteogenic differentiation media supplemented with BMP-2 (OM + BMP2). Bars represent mean ± SD of six donors. *p < 0.05 as compared to the respective sample cultured with OM. ^#,$^*p* < 0.05 as compared to the respective sample in P3.

The original Article and accompanying Supplementary Information files have been corrected.

