# Author Correction: Osteogenic differentiation of human mesenchymal stromal cells and fibroblasts differs depending on tissue origin and replicative senescence

**DOI:** 10.1038/s41598-021-95755-4

**Published:** 2021-08-09

**Authors:** Vera Grotheer, Nadine Skrynecki, Lisa Oezel, Joachim Windolf, Jan Grassmann

**Affiliations:** Clinic for Orthopedics and Trauma Surgery, Medical Faculty of the Heinrich Heine University, Moorenstr. 5, 40225 Düsseldorf, Germany

Correction to: *Scientific Reports* 10.1038/s41598-021-91501-y, published online 07 June 2021

The original version of this Article contained an error in the order of the author names, which was incorrectly given as Vera Grotheer, Nadine Skrynecki, Lisa Oezel, Jan Grassmann & Joachim Windolf.

The original Article has been corrected.

