# Supplementary material for: Osteogenic differentiation of human mesenchymal stromal cells and fibroblasts differs depending on tissue origin and replicative senescence
Source: Sci Rep. 2021 Jun 7;11:11968. doi: 10.1038/s41598-021-91501-y (PMC8184777; doi:10.1038/s41598-021-91501-y)

Osteogenic differentiation of human mesenchymal stromal cells and fibroblasts differs depending on tissue origin and replicative senescence

Vera Grotheer[1], Nadine Skrynecki[1], Lisa Oezel[1], Joachim Windolf[1], Jan Grassmann[1]

[1]Clinic for Orthopedics and Trauma Surgery, Medical Faculty of the Heinrich Heine University, Düsseldorf, Germany

Correspondence to:

Vera Grotheer

Clinic for Orthopedics and Trauma Surgery

Medical Faculty of the Heinrich Heine University

Moorenstr. 5

40225 Düsseldorf

Germany

E-mail: vera.grotheer@med.uni-duesseldorf.de

Supplement 1

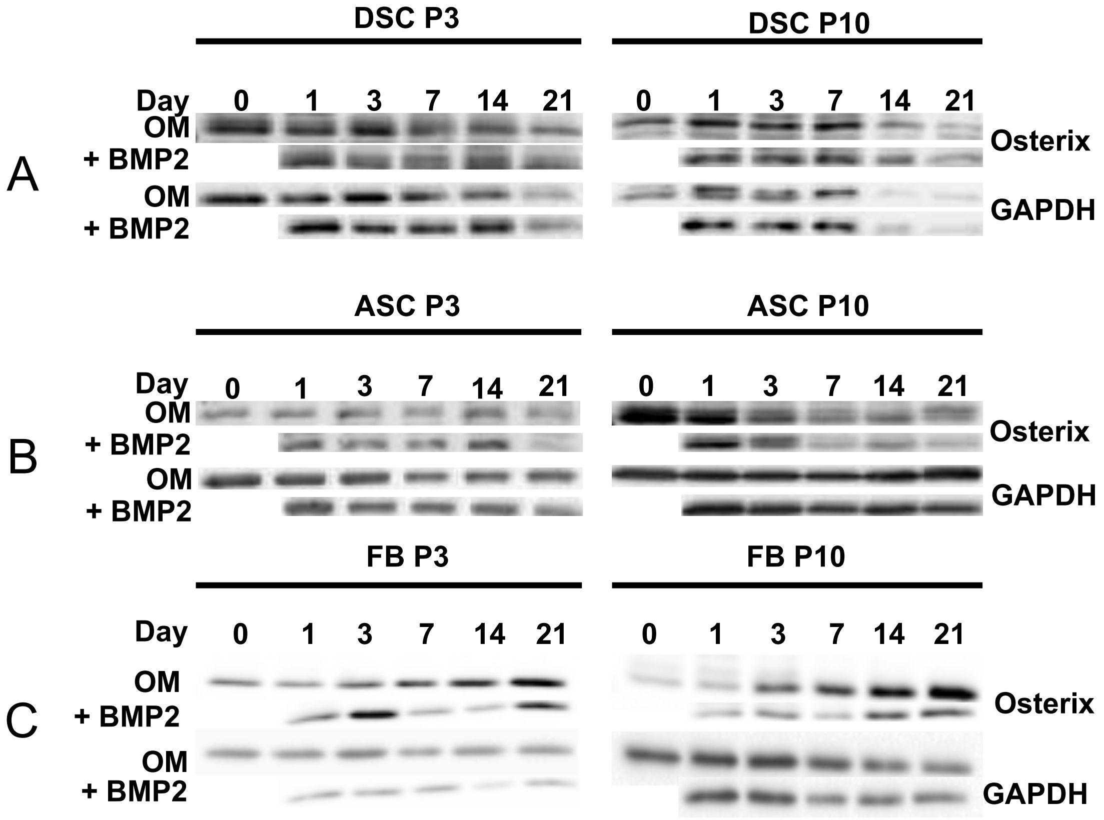

LEGENDS TO THE SUPPLEMENTS

Protein analysis of Osterix in DSC, ASC, and FB

DSC, ASC and FB were differentiated osteogenically with or without BMP-2. At day 0, 1, 3, 7 14, and 21 proteins were harvested, and Western Blot Analysis of Osterix and as a house keeping gene GAPDH were performed. Exemplary cropped Western Blots were presented. Full-length blots are presented in Supplementary Figure 4. **A** Osterix protein expression of DSC in P3 (left) and in P 10 (right) were demonstrated. Osterix protein expression declined in the course of differentiation. **B** Osterix protein expression of ASC in P3 (left) and in P 10 (right) were demonstrated. Osterix protein expression declined in the course of differentiation. **C** Osterix protein expression of FB in P3 (left) and in P 10 (right) were demonstrated. Osterix protein expression increased in P3 treated with OM and in P 10 in the course of differentiation.

Supplement 2

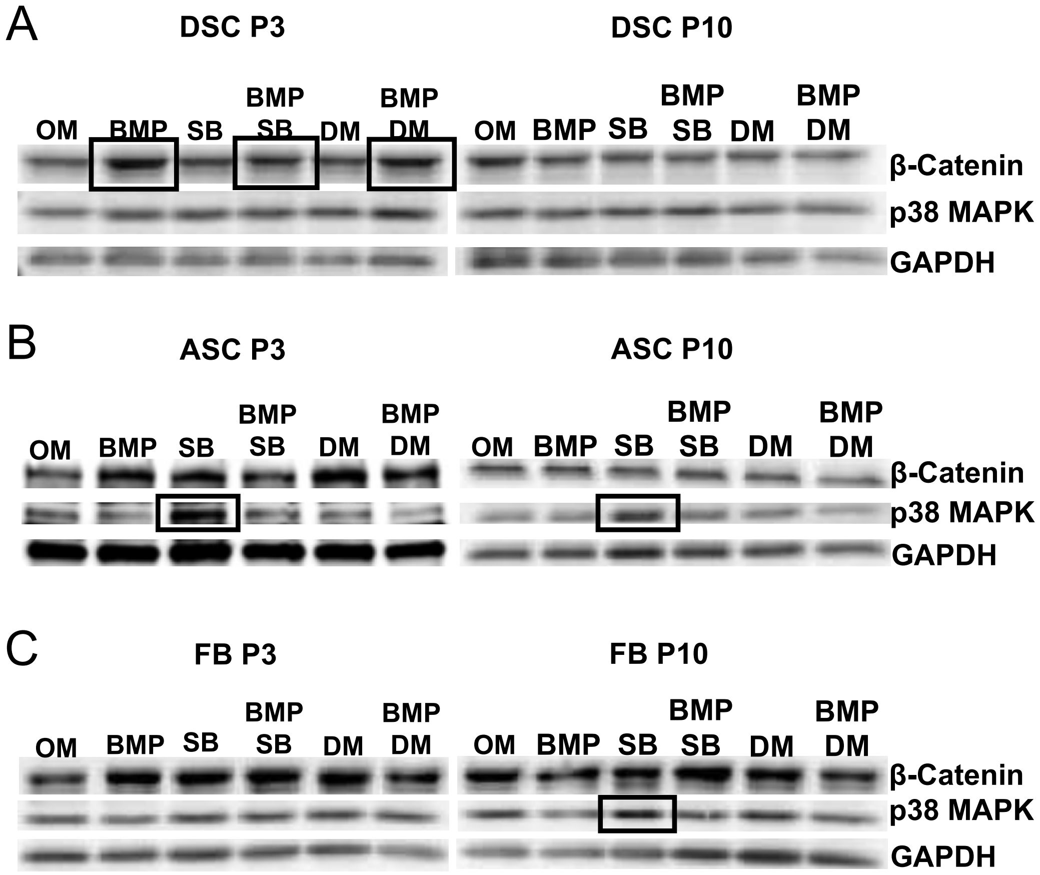

Protein analysis of β-catenin and MAPK (p38) in DSC, ASC, and FB

DSC, ASC, and FB were treated in the following order: with osteogenic differentiation (OM) media either supplemented with BMP-2 (BMP), or with SB431542 (SB), or with SB431542 + BMP-2 ($\frac{BMP}{SB}$), or dorsomorphin (DM), or and BMP-2 + dorsomorphin ($\frac{BMP}{DM}$). After seven days proteins were harvested and Western Blot analysis were performed and as a housekeeping gene GAPDH was used. Respective cropped Western Blot analysis of P 3 (left) and P 10 (right) were exemplary presented. Full-length blots are presented in Supplementary Figure 5. **A** β-catenin and MAPK (p38) expression in DSC. β-catenin expression increased in P 3, whenever DSC were treated with BMP-2. **B** β-catenin and MAPK (p38) expression in ASC. Especially in ASC treated with OM and SB431542 the p38 protein expression was elevated in P 3 and P 10 as the osteogenic differentiation potential was. **C** β-catenin and MAPK (p38) expression in FB. In FB in P 10 treated with OM and SB431542 the p38 protein expression was elevated.

Supplement 3

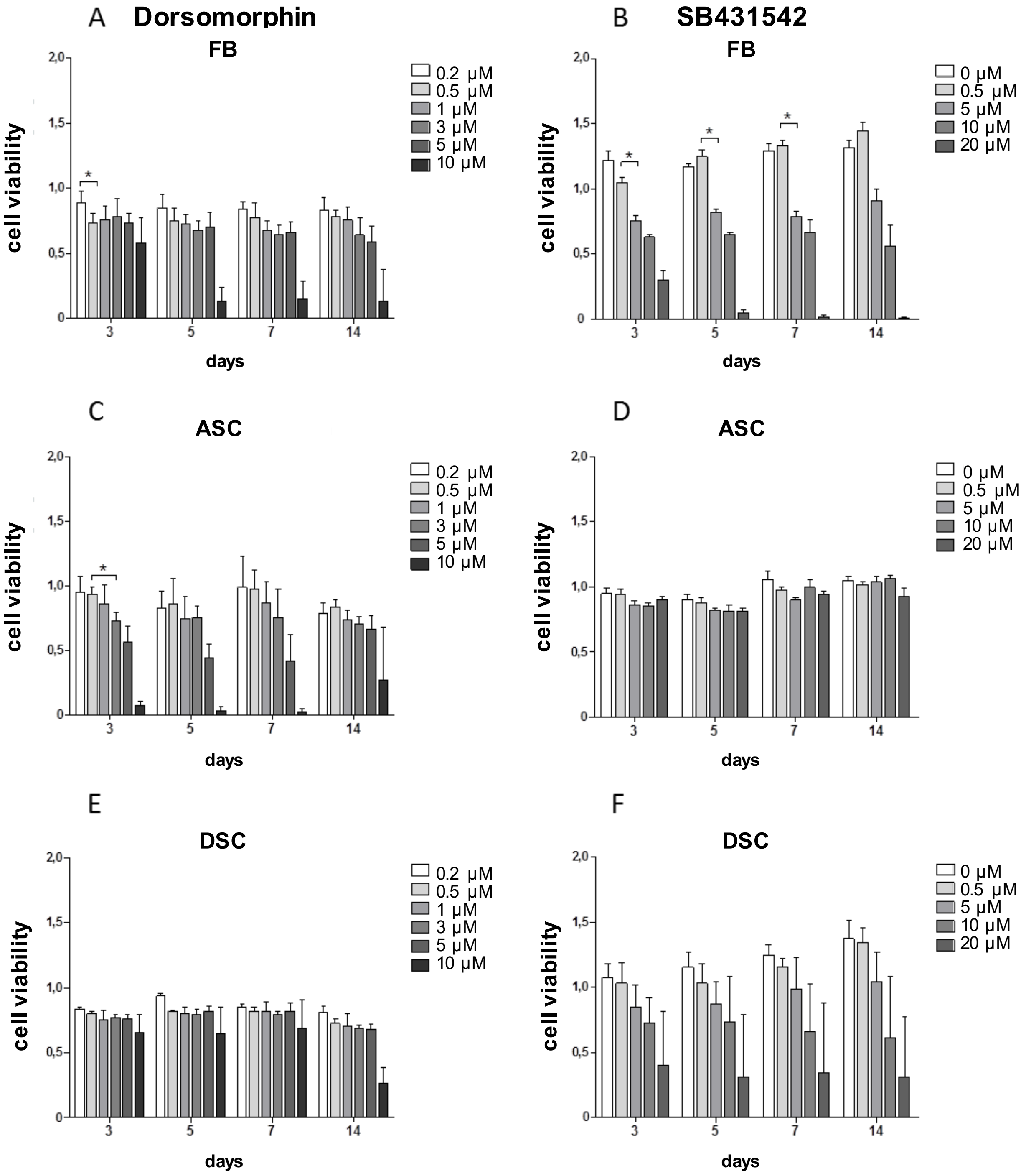

Evaluation of optimal dorsomorphin and SB431542 concentration for osteogenic differentiation.

A Cell viability analysis of FB after treatment with dorsomorphin **B** Cell viability analysis of FB after treatment with SB431542 **C** Cell viability analysis of ASC after treatment with dorsomorphin **D** Cell viability analysis of ASC after treatment with SB431542 **E** Cell viability analysis of DSC after treatment with dorsomorphin **F** Cell viability analysis of ASC after treatment with SB431542.

Bars represent mean ± SD of three individual experiments.

Full length Blots

Supplements 4

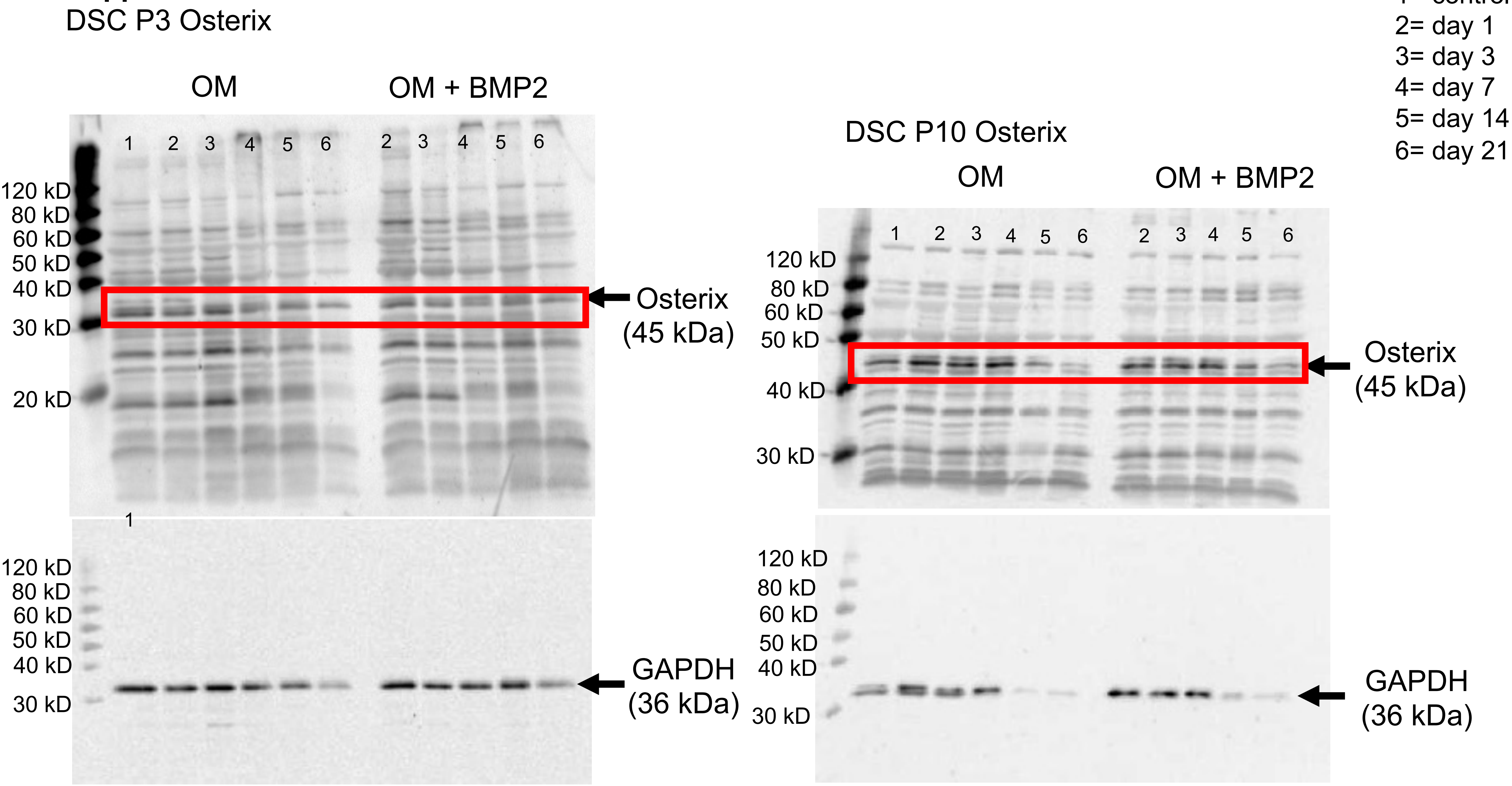

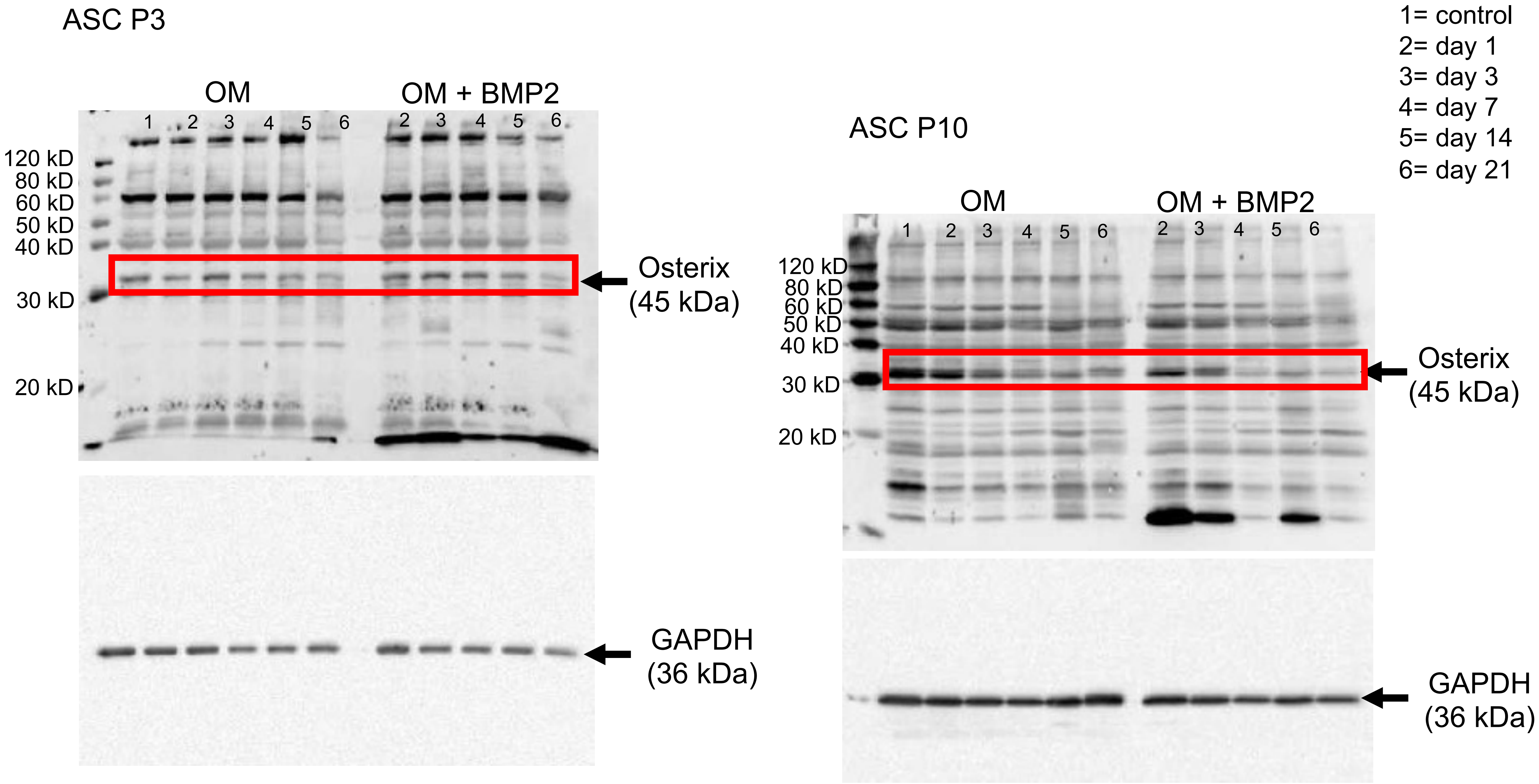
ASC P3
OM
OM + BMP2
1 2 3 4 5 6
2 3 4 5 6
120 kD
80 kD
60 kD
50 kD
40 kD
30 kD
20 kD
Osterix (45 kDa)
GAPDH (36 kDa)
ASC P10
OM
OM + BMP2
1 2 3 4 5 6
2 3 4 5 6
120 kD
80 kD
60 kD
50 kD
40 kD
30 kD
20 kD
Osterix (45 kDa)
GAPDH (36 kDa)
1= control
2= day 1
3= day 3
4= day 7
5= day 14
6= day 21

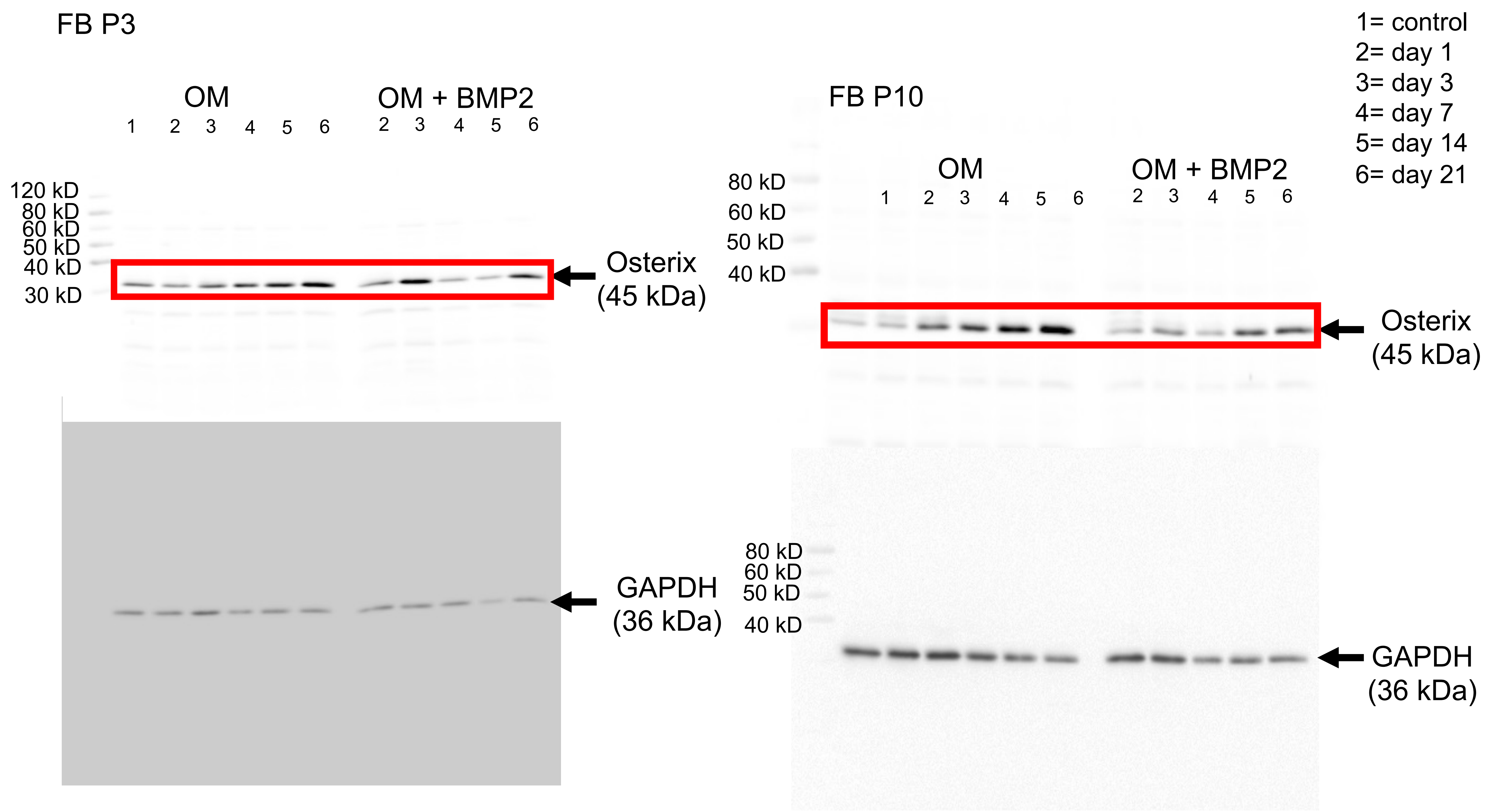
FB P3
OM
OM + BMP2
1 2 3 4 5 6
2 3 4 5 6
120 kD
80 kD
60 kD
50 kD
40 kD
30 kD
Osterix (45 kDa)
GAPDH (36 kDa)
FB P10
OM
OM + BMP2
1 2 3 4 5 6
2 3 4 5 6
80 kD
60 kD
50 kD
40 kD
Osterix (45 kDa)
80 kD
60 kD
50 kD
40 kD
GAPDH (36 kDa)
1= control
2= day 1
3= day 3
4= day 7
5= day 14
6= day 21

Supplements 5
DSC

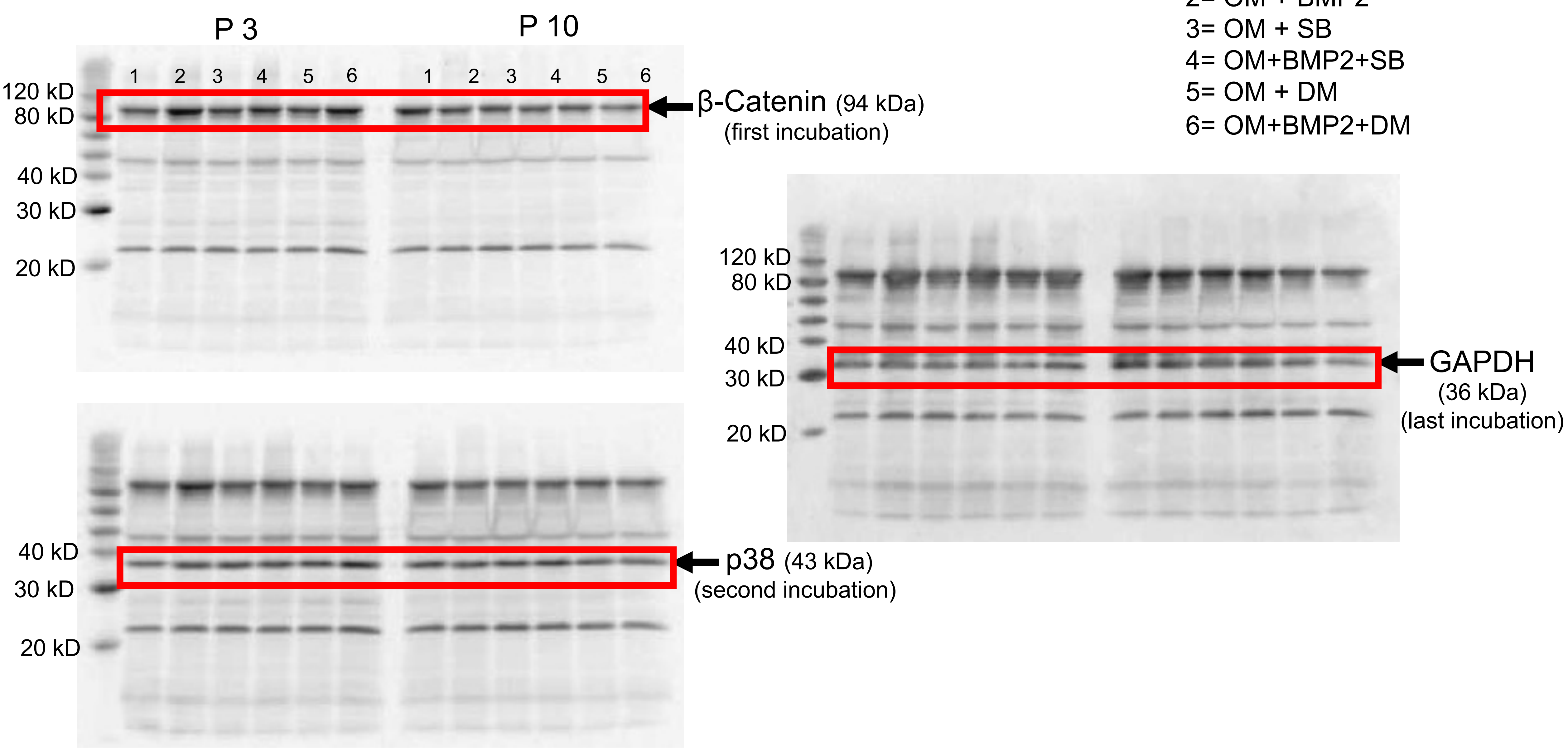

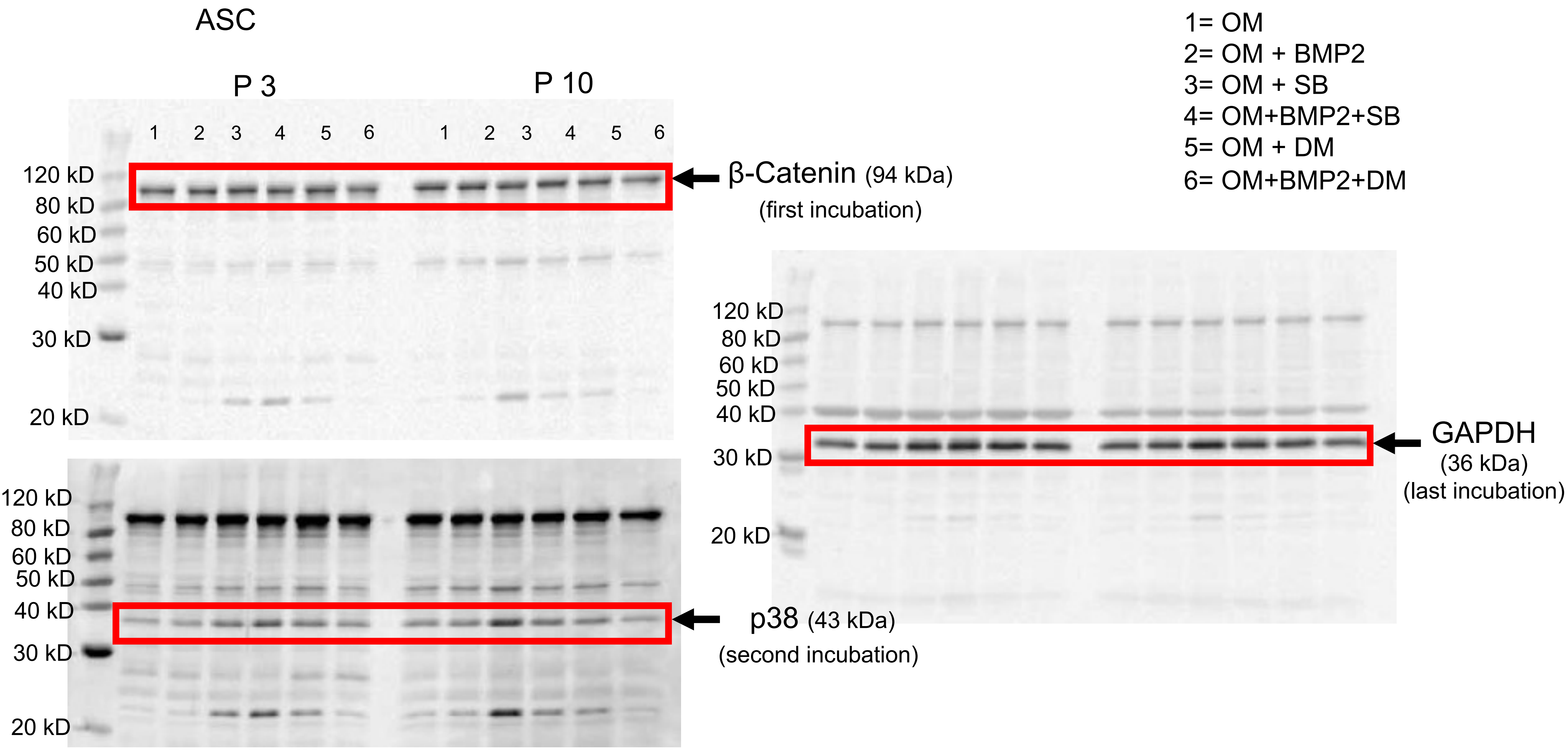
ASC
P 3
P 10
1 2 3 4 5 6 1 2 3 4 5 6
120 kD
80 kD
60 kD
50 kD
40 kD
30 kD
20 kD
β-Catenin (94 kDa)
(first incubation)
p38 (43 kDa)
(second incubation)
GAPDH
(36 kDa)
(last incubation)
1= OM
2= OM + BMP2
3= OM + SB
4= OM+BMP2+SB
5= OM + DM
6= OM+BMP2+DM

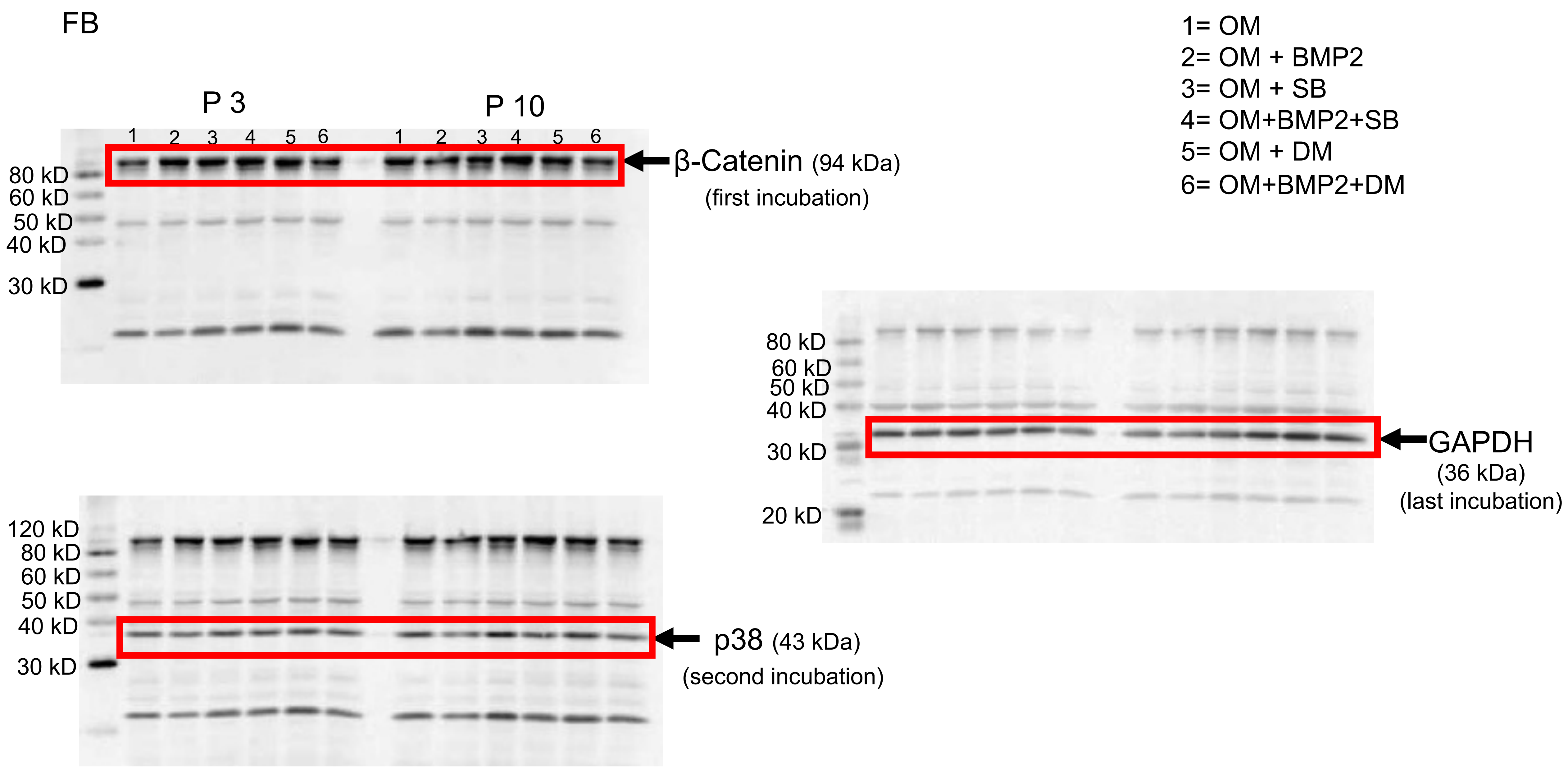
FB
1= OM
2= OM + BMP2
3= OM + SB
4= OM+BMP2+SB
5= OM + DM
6= OM+BMP2+DM
P 3
P 10
1 2 3 4 5 6 1 2 3 4 5 6
80 kD
60 kD
50 kD
40 kD
30 kD
β-Catenin (94 kDa)
(first incubation)
80 kD
60 kD
50 kD
40 kD
30 kD
20 kD
GAPDH
(36 kDa)
(last incubation)
120 kD
80 kD
60 kD
50 kD
40 kD
30 kD
p38 (43 kDa)
(second incubation)

Supplements 5

β-galactosidase Western Blot

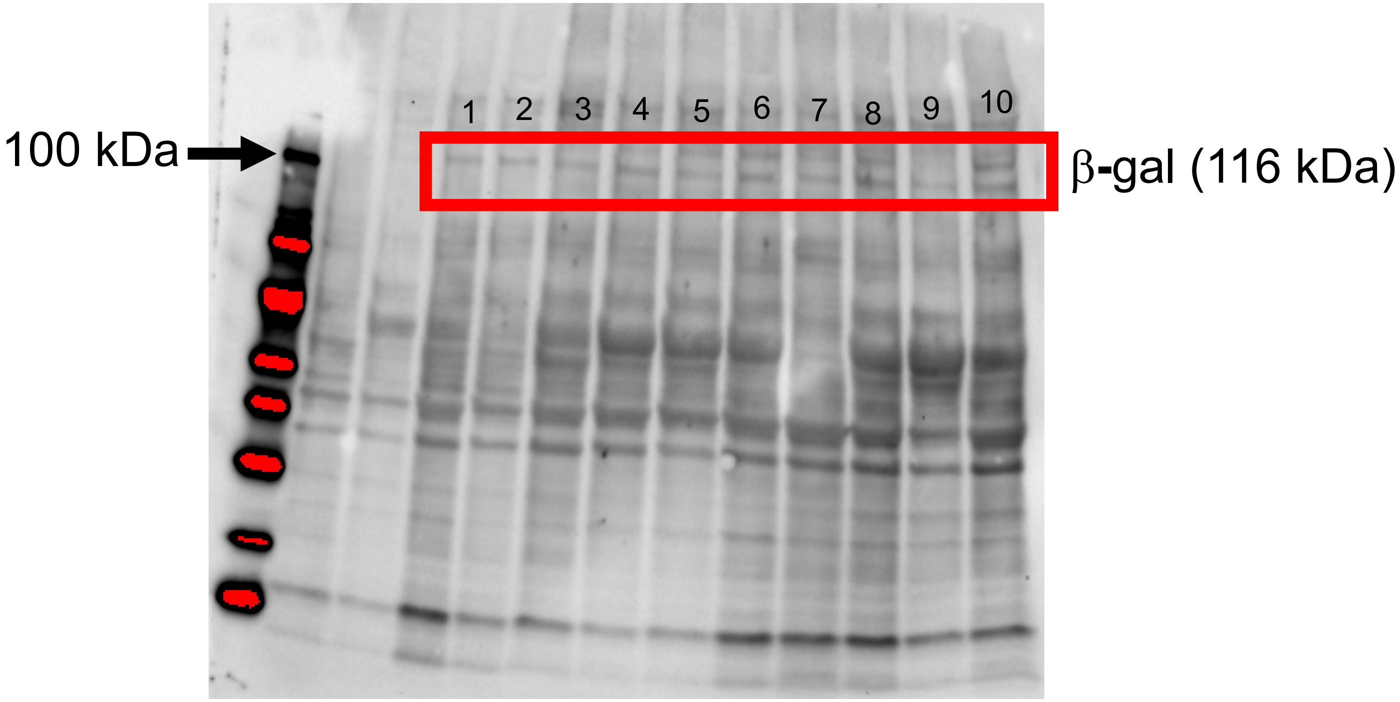

1 = DSC P3
2 = DSC P10
3 = ASC P3
4 = ASC P10
5 = ASC P3
6 = ASC P10
7 = FB P3
8 = FB P10
9 = FB P3
10 = FB 10

Normalisation Channel

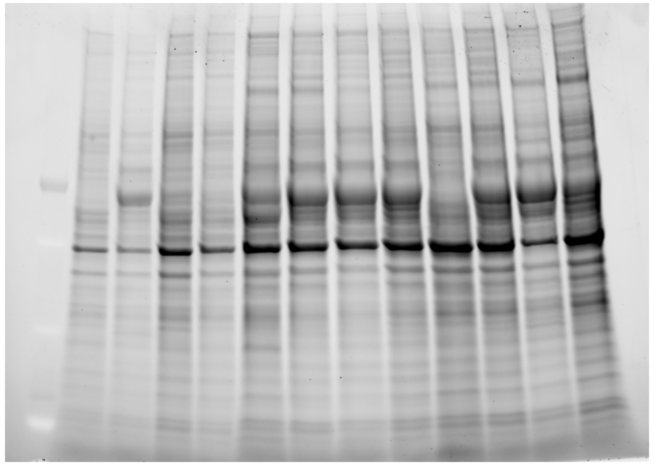

Supplement: Supplementary file 1 — Supplementary Information. [file 41598_2021_91501_MOESM1_ESM.pdf]
